# The interaction of tPA with NMDAR1 drives neuroinflammation and neurodegeneration in α-synuclein-mediated neurotoxicity

**DOI:** 10.1186/s12974-025-03336-3

**Published:** 2025-01-14

**Authors:** Daniel Torrente, Enming J. Su, Alí Francisco Citalán-Madrid, Gerald P. Schielke, Daniel Magaoay, Mark Warnock, Tamara Stevenson, Kris Mann, Flavie Lesept, Nathalie Delétage, Manuel Blanc, Erin H. Norris, Denis Vivien, Daniel A. Lawrence

**Affiliations:** 1 Department of Molecular and Integrative Physiology, University of Michigan Medical School, Ann Arbor, MI USA; 2 Patricia and John Rosenwald Laboratory of Neurobiology and Genetics, The Rockefeller University, New York, NY USA; 3 Department of Internal Medicine, Division of Cardiovascular Medicine, University of Michigan Medical School, 7301 MSRB III, 1150 W. Medical Center Dr., Ann Arbor, MI 48109-0644 USA; 4 Lys Therapeutics, Main offices: 56 rue Saint Jean de Dieu, Lyon, 69007 France; 5 Lys Therapeutics, HQ: Cyceron, Boulevard Henri Becquerel, Caen, 14000 France; 6 Physiopathology and Imaging of Neurological Disorders (PhIND), UNICAEN, INSERM, GIP Cyceron, Institut Blood and Brain @Caen-Normandie (BB@C), UMR-S U1237, Normandie Univ, Caen, France; 7 Department of Clinical Research, Caen University Hospital, CHU, Caen, France

**Keywords:** tPA, α-synuclein, Substantia nigra, Parkinson’s disease, Neuroinflammation, Dopaminergic neurons, NMDAR, Glunomab, Microglia, T-cell

## Abstract

The thrombolytic protease tissue plasminogen activator (tPA) is expressed in the CNS, where it regulates diverse functions including neuronal plasticity, neuroinflammation, and blood-brain-barrier integrity. However, its role in different brain regions such as the substantia nigra (SN) is largely unexplored. In this study, we characterize tPA expression, activity, and localization in the SN using a combination of retrograde tracing and β-galactosidase tPA reporter mice. We further investigate tPA’s potential role in SN pathology in an α-synuclein mouse model of Parkinson’s disease (PD). To characterize the mechanism of tPA action in α-synuclein-mediated pathology in the SN and to identify possible therapeutic pathways, we performed RNA-seq analysis of the SN and used multiple transgenic mouse models. These included tPA deficient mice and two newly developed transgenic mice, a knock-in model expressing endogenous levels of proteolytically inactive tPA (tPA Ala-KI) and a second model overexpressing proteolytically inactive tPA (tPA Ala-BAC). Our findings show that striatal GABAergic neurons send tPA^+^ projections to dopaminergic (DA)-neurons in the SN and that tPA is released from SN-derived synaptosomes upon stimulation. We also found that tPA levels in the SN increased following α-synuclein overexpression. Importantly, tPA deficiency protects DA-neurons from degeneration, prevents behavioral deficits, and reduces microglia activation and T-cell infiltration induced by α-synuclein overexpression. RNA-seq analysis indicates that tPA in the SN is required for the upregulation of genes involved in the innate and adaptive immune responses induced by α-synuclein overexpression. Overexpression of α-synuclein in tPA Ala-KI mice, expressing only proteolytically inactive tPA, confirms that tPA-mediated neuroinflammation and neurodegeneration is independent of its proteolytic activity. Moreover, overexpression of proteolytically inactive tPA in tPA Ala-BAC mice leads to increased neuroinflammation and neurodegeneration compared to mice expressing normal levels of tPA, suggesting a tPA dose response. Finally, treatment of mice with glunomab, a neutralizing antibody that selectively blocks tPA binding to the N-methyl-D-aspartate receptor-1 (NMDAR1) without affecting NMDAR1 ion channel function, identifies the tPA interaction with NMDAR1 as necessary for tPA-mediated neuroinflammation and neurodegeneration in response to α-synuclein-mediated neurotoxicity. Thus, our data identifies a novel pathway that promotes DA-neuron degeneration and suggests a potential therapeutic intervention for PD targeting the tPA-NMDAR1 interaction.

**Supplementary Information:**

The online version contains supplementary material available at 10.1186/s12974-025-03336-3.

## Background

The serine protease tissue plasminogen activator (tPA) is well known for its role in fibrinolysis [1]. Beyond the fibrinolytic system, tPA expression is also reported in the CNS, where it regulates different brain functions independent of fibrinolysis, including neuronal plasticity, neuroinflammation, and blood-brain-barrier integrity [2–4]. In neurons, tPA is stored and released by axon terminals in response to neuronal depolarization [5, 6], and evidence suggests that tPA is rapidly trafficked from neuronal cell bodies to axons [7, 8], making it challenging to detect in cell bodies and to track brain circuits involving tPA projections.

Since the first reports of tPA expression in the CNS and its association with neuronal activity [9, 10], significant progress has been made in understanding tPA function in brain physiology and pathology [4, 6, 11]. However, the characterization of tPA in the CNS has primarily focused on tPA-rich structures such as the hippocampus, amygdala, cerebellum, and cortex [10, 12–16], while tPA expression and function in other brain regions is largely unexplored.

We recently identified tPA expression in the substantia nigra (SN) [8, 17]. The SN is extensively studied for its involvement in Parkinson’s disease (PD), the second most common neurodegenerative disease worldwide. PD is characterized by the progressive degeneration of dopaminergic (DA) neurons in the SN *pars compacta* (SNpc) and by the formation of protein aggregates primarily composed of α-synuclein (α-SYN) [18]. Despite the unknown cause(s) of sporadic PD, many factors that drive disease progression have been identified [19–21]. Microglia-induced neuroinflammation and T-cell-mediated neurotoxicity are suggested to play a significant role in DA-neuron degeneration in both animal models and human disease [19, 20, 22, 23]. However, the molecular mechanism(s) involved in neuroinflammation in the initial stages of DA-neuron degeneration in the SN are poorly understood. Therefore, identifying pathways that drive inflammation and neurodegeneration in the SN could provide insight into the development of disease-modifying therapies in PD.

tPA is linked to neuronal cell death in different models of CNS pathologies including stroke, multiple sclerosis, and seizures [24–26]. These studies suggest that tPA can promote pro-inflammatory and neurotoxic events via either proteolytic-dependent or proteolytic-independent mechanisms [24, 26–28]. In particular, the interaction between tPA and the N-methyl-D-aspartate receptor (NMDAR) was reported to increase pro-inflammatory markers in stroke [29] and the infiltration of macrophages and T-cells, in a model of multiple sclerosis [28]. Characterization of tPA in the SN and its possible role in SN pathologies, such as those observed in PD, has not been explored. Based on the observation that tPA is present in the SN and previous reports linking tPA with neuroinflammatory and neurotoxic events, this study focused on characterizing tPA expression in the SN and exploring its potential role in neuroinflammation and DA-neuron degeneration in an α-synuclein mouse model of PD, aiming to identify novel therapeutic pathways for PD treatment.

## Materials and methods

### Animal models

Male and female mice between 8 and 18 weeks-of-age were used. C57BL/6J mice and WDFY-1 KO mice were obtained from Jackson Laboratories. tPA-KO [30], neuroserpin (Nsp)-KO [31], and plasminogen activator inhibitor-1 (PAI-1)-KO [32] mice were backcrossed at least 10 generations into C57BL/6J WT mice. These mice were then used to generate tPA/WDFY-1 and Nsp/PAI-1 double-KO mice. tPA Ala-KI mice were generated in collaboration with Innovative Research, Inc Novi MI. This mouse expresses endogenous levels of proteolytically-inactive tPA and was generated in a C57BL/6J background using CRISPER technology targeting the active site of tPA, mutating Serine 510 to an Alanine in exon 14 of the *PLAT* (tPA) gene (Ser510Ala; available at Innovative Research; IGMSKITPANPNP). The tPA Ala-BAC mice were generated at the University of Michigan Transgenic core. tPA Ala-BAC mice overexpress proteolytically inactive tPA driven by the endogenous regulatory elements of tPA. In these mice, tPA Ala (Ser510Ala) is fused to the cerulean fluorescent protein (CFP) in the C-terminus of exon 14 of the tPA gene. This mouse was generated using a bacterial artificial chromosome (BAC), and tPA Ala overexpression was achieved by multiple insertions of the tPA Ala-BAC construct as previously described [8]. The tPA Ala-BAC construct containing the regulatory sequences of tPA, the Ser510Ala point mutation, and the cerulean sequence was microinjected into eggs from C57BL/6 x SJL F1/TAC female mice. tPA Ala-BAC mice were backcrossed at least 10 generations into C57BL/6J WT mice and then crossed with tPA-KO mice. This procedure was necessary to obtain tPA Ala-BAC mice expressing only proteolytically inactive tPA without expressing WT tPA. In-gel zymography and CFP presence were used to confirm tPA Ala-BAC genotype. The *Plat*βGAL (tPAβGAL) reporter mice were obtained from UC Davis Knockout Mouse Project Repository (Project ID: VG15085) and were previously characterized [8]. Mice were housed under a 12-hour light/dark cycle with free access to water and standard rodent chow. All animal procedures were approved by and carried out in accordance with the guidelines of the Institutional Animal Care and Use Committee at the University of Michigan.

### rAAV2-hα-SYN design and production

Recombinant adeno-associated virus serotype 2 (rAAV2) expressing human wild-type α-SYN (rAAV2-hα-SYN) or rAAV2-empty (rAAV2-control) virus were produced at the University of Michigan Vector core as previously described [33]. Cloning of hα-SYN was done using the pAAV-hα-SYN WT plasmid as a template (#36055, Addgene) and the pAAV-CBA plasmid as the vector backbone (#81008; Addgene). The resulting plasmid pAAV-CBA-hα-SYN was used to produce rAAV2-hα-SYN virus, where hα-SYN expression is driven by the chicken-beta actin (CBA) promoter. A rAAV2-control virus was generated alongside the rAAV2-hα-SYN virus. The rAAV2-empty virus was generated using a pCWB-CBA-empty plasmid which does not express any protein. This plasmid was produced by excising the eGFP cassette from a pCWB-CBA-GFP plasmid (in house). The final titers of the viral stocks were determined by qPCR and ranged between 2.0 × 10^13^ and 3.12 × 10^13^ vg/mL (plasmids and viruses are available at the University of Michigan Vector core) [33]. No significant differences in dopaminergic neuron reduction were observed between rAAV2-empty and rAAV2-eGFP virus injections (Fig. S1A). However, rAAV2-eGFP virus injection in WT mice showed a trend toward dopaminergic neuron degeneration compared to rAAV2-empty virus. Importantly, rAAV2-hα-SYN induced significantly greater dopaminergic neuron degeneration compared to both rAAV2-empty and rAAV2-eGFP 4 weeks after intranigral virus injections. Accordingly, and consistent with other studies [34], we have used the rAAV2-empty virus as a control in our experiments.

### Stereotaxic rAAV2- hα-SYN injection in SN

Male and female mice between 8 and 18 weeks old were used for recombinant adeno-associated virus 2 (rAAV2)-hα-SYN injection as previously described [33]. Briefly, mice were anesthetized with 2% isoflurane and secured on a stereotactic frame. WT, tPA-KO, tPA Ala-KI, tPA Ala-BAC, WDFY-1-KO, and WDFY-1/tPA-KO mice received a unilateral injection of rAAV2-hα-SYN at a final concentration of 2 × 10^13^ viral genome copies per milliliter (vg/mL) into the SN. rAAV2-control was injected in the SN of WT and tPA-KO mice at the same concentration. The SN coordinates used for stereotaxic injection was AP= -3.1, ML=-1.4, DV = 4.2 mm. A total of 2 μL of rAAV2-hα-SYN or rAAV2-control virus was injected at a rate of 0.25 μL/min with a 33G nanofil blunt needle (NF33BL-2, World Precision Instruments) connected to FEP tubing (cma3409501, Harvard apparatus) and a 25 uL Hamilton syringe. After virus infusion, the nanofil blunt needle was left in place for 5 min. Consistent with previous reports in this PD model [35], we found that injection of the rAAV2-hα-SYN resulted in increased α-SYN phosphorylation of serine 129 (pS129-SYN; Fig. S1B, C) in dopaminergic neurons, indicating the formation of α-SYN protein aggregates. We also did not observe any influence of sex on the outcome of experiments (Fig. S1D-G), therefore, results from males and females were combined for data analysis. A total of 4 mice were excluded from analysis due to inaccurate virus injection into the SN. Four weeks after virus injection, mice were perfused with PBS and 4% PFA for immunohistochemical analysis, or the brains were quickly harvested without perfusion and the injected and uninjected SN were dissected out for RNA isolation. The 4-week time point after rAAV2-hα-SYN injection was selected because previous research, including our own, shows that this early stage is marked by a significant increase in neuroinflammation and DA-neuron degeneration in the SN [33].

### Retrograde tracing

For retrograde tracing experiments in the SN, mice were anesthetized with 2% isoflurane, secured on a stereotactic frame, and Cholera toxin-B (CTB)-488 at a concentration of 0.1% in 1X PBS (C34775, Invitrogen) was injected into the SN of PlatβGAL^+/−^ or PlatβGAL^−/−^ (control) mice. A total of 300 nL was delivered into the SN at a rate of 0.1μL/min, and after CTB-488 infusion, the needle was left in place for 5 min. The SN coordinates used for stereotaxic injection: AP= -3.1, ML=-1.4, DV = 4.2 mm. All injections were done in the left hemisphere. One week after injection, mice were PBS and 2% PFA perfused (light fixation). Harvested brains were dehydrated in 30% sucrose for 2–3 days and incubated in OCT. Coronal sections (30 μm-thick) sampling the whole brain were analyzed for beta-galactosidase activity and immunohistochemical analysis.

### β-galactosidase assay

Coronal brain sections sampling the whole brain (30 μm-thick) from tPAβGAL^+/−^ and tPAβGAL^−/−^ (control) mice 1 week after CTB-488 injection in the SN were used for LacZ reporter gene expression using the β-Galactosidase Reporter Gene Staining kit following manufacture instructions (GALS, Sigma).

### Immunohistochemistry

For immunofluorescence, mice were perfused with PBS and 4% PFA. Brains were then harvested, post-fixed in PFA, dehydrated in 30% sucrose, and embedded in OCT. Frozen sections (14 μm-thick) were permeabilized and blocked in 0.5% TritonX-100 and 5% bovine serum albumin in 0.5% TritonX-100/1X PBS, respectively. Sections were incubated with primary antibodies in blocking solution overnight at 4 °C followed by incubation with 488, 564, or 647 Alexa-Fluor conjugated secondary antibodies for 1 h at room temperature. After secondary antibody incubations, sections were washed with 0.1% NP-40/1X PBS. DAPI was used to detect cell nuclei. Sections were mounted using ProLong™ Diamond Antifade Mountant (P36961; Life Technologies). The primary antibodies used were: anti-TH (1:1000; ab113, Abcam), anti-hα-SYN (1:300; ab138501, Abcam), anti-tPA (12 ug/mL; ASMTPA-GF-HT, Innovative Research), anti-TMEM119 (1:500; ab209064, Abcam), anti-MHC-I (1:200; NB100-64952, Novus), anti-CD16/32 (1:100; #101301, BioLegend), anti-C1q (1:500; ab182451, Abcam), anti-Podocalyxin (PODO; 1:150; AF1556, R&D Systems), anti-DARPP-32 (1:500; 382 004, SySy), anti-VAMP-2 (1:200; 104 204, SySy), anti-VGAT (1:250; 131 004, SySy), anti-Vglut1 (1:200; AB5905, Chemicon), anti-Vglut2 (1:200; 135 404, SySy), anti-CD3 (1:200; 100347, BioLegend), anti-CD8 (1:200; 14-0081-82, eBioscience), anti-CD4 (1:200; 14-0041-81, eBioscience), anti-pS129-SYN (1:500; 23706, Cell Signaling), and anti-NMDAR1 (1:100; AGP-046, Alomone Labs). For staining involving tPA, DARPP-32 and hα-SYN, heat-mediated antigen retrieval was performed using DAKO retrieval solution (S1700; DAKO). All images were acquired with a Nikon Ti-E Eclipse Microscope using a 20x objective (Plan-Apo, 0.75 numerical aperture) or a 60x oil objective (Plan-Apo, 1.4 numerical aperture). Confocal images were acquired using CREST X-Light V2 Spinning Disk. Z-stacks of 60x images were collected at 0.5 μm increments with a total thickness of ∼ 6 μm. Images were captured with an ORCA-fusion camera (C14440-20UP, Hamamatsu) or an Andor Zyla camera (Zyla 4.2 sCMOS, Oxford Instruments). Large scan function with Z step focus was used to capture stitched images of the SN using a 20x objective. Confocal images are shown in orthogonal view depicting colocalization in the xy (center), xz (bottom), and yz (right) planes or as maximum intensity projection images. Images are representative of the respective staining and were processed and analyzed using NIS-elements advanced research software (Nikon Instrument Inc) and FIJI-Image J open software [36].

### DA-neurons, fluorescence intensity and colocalization quantification

Estimation of DA-neuron degeneration in the SN was performed by counting the total number of TH^+^ neurons in the injected and uninjected SN 4 weeks after rAAV2-hα-SYN or rAAV2-control virus injection as previously described [33]. Coronal brain sections. (14 μm-thick) were sampled at intervals of 112 μm through the rostrocaudal extent of the SN, and at least 4 sections per mouse were counted. The total number of TH^+^ neurons (Nt) in the injected and uninjected hemisphere of the SN were estimated using model-based stereology incorporating the Abercrombie correction with the following formula: Nt = Ns × (St/Ss) × M/(M + D), where Ns is the total number of neuron counted, St is the total number of sections in the brain region, Ss is the number of sections sampled, M is the thickness of the section, and D is the average diameter of the counted neurons [33, 37]. The average diameter of TH^+^ neurons was calculated averaging the min and max Feret diameter. Nt for the uninjected SN was used as an internal control in every mouse and a % of DA-neuron survival was obtained using the following formula: TH^+^ neuron survival (%) = (Nt_injected SN_/ Nt_uninjected SN_) x 100. The genotype and treatments of mice were unknown to the investigator at the time of quantification.

Fluorescence intensity of tPA, hα-SYN, C1q, MHC-I, and CD16/32 in the injected and uninjected SN 4 weeks after rAAV2-hα-SYN or rAAV2-control virus injection was performed as previously described [33]. Coronal brain sections (14 μm-thick) co-stained with TH and hα-SYN, C1q, MHC-I, or CD16/32 were sampled in intervals of 112 μm through the rostrocaudal extent of the SN; 4 sections per mouse were quantified. A region of interest defining the entire SN *pars compacta* (SNpc) using TH co-staining was used to quantify the sum fluorescence intensity above background in the injected and uninjected SN. A relative fluorescence value was calculated in each mouse using the uninjected SN as an internal control. For the average number of CD3^+^ cells quantification in the SN 4 weeks after rAAV2-hα-SYN injection, 14 μm-thick coronal brain sections were stained with CD3, TH, and CD4 or CD8. The average number of CD3^+^ cells associated with the SNpc were counted by defining the SNpc using TH staining and the average number of CD3^+^ cells were then estimated in the injected and uninjected (rarely observed) SNpc in 4 sections per mouse sampling the rostrocaudal extent of the SN. To estimate the % of CD3^+^ CD4^+^ and CD3^+^ CD8^+^ T-cells infiltrating the SNpc we use the following formula: % of CD4 or CD8 T-cells= (_total_CD3^+^ CD(4^+^ or 8^+^) T-cells/ _total_CD3^+^ T-cells) x 100.

For colocalization analysis of tPA in the SN, deconvoluted confocal images of tPA co-stained with VAMP-2, VGAT, Vglut1 or Vglut2 were used. Deconvolution was performed in 60x images in NIS-elements advanced research software using the Landweber algorithm. Mander’s overlap coefficient (MOC) and Costes significant test were calculated in Z-stacks of 60x images (0.5 μm step; total thickness of ∼ 6 μm) via the Coloc2 algorithm in FIJI-ImageJ using the bisection threshold regression. At least two randomly selected regions of interest were analyzed per image and a total of 3 sections per mouse were used to calculate the average MOC above threshold between tPA and VAMP-2, VGAT, Vglut 1 or Vglut 2. MOC values range from 0 to 1, where 0 is non-overlapping pixels and 1 is the absolute colocalization between pixels. MOC values in this study represent tPA pixel colocalization with VAMP-2, VGAT, Vglut1, or Vglut2 pixels. In all colocalization analysis, Costes significant test confirmed that the observed colocalization was not due to random events. For colocalization analysis of MHC-I and CD16/32 with TMEM119 in WT mice after rAAV2-hα-SYN injection in the SN, confocal images of MHC-I or CD16/32 co-stained with TMEM119 were used. Denoised and colocalization calculations were performed in 60x images in NIS-elements advanced research software. MOC was calculated in Z-stacks of 60x images (0.5 μm step; total thickness of ∼ 6 μm). At least 3 sections per mouse were used to calculate the average MOC above threshold between MHC-I or CD16/3 with TMEM119.

### Western blot and Luminex assay

Western blots of protein extracts of the SN, hippocampus, and crude synaptosome preparation from the SN were carried out as follows: Samples were boiled for 7 min at 96 °C in non-reduced conditions and loaded in a 4–15% precast SDS gel (4561084; Bio-Rad). Proteins were transfer overnight to a nitrocellulose membrane at 4 °C. Membranes were then blocked at RT for 1 h using Intercept Blocking buffer (927-60001; LI-COR). All primary antibodies were incubated overnight at 4 °C in blocking buffer. The primary antibody used were: anti-actin (1:1000; A2066, Sigma), anti-TH (1:1000; ab112, Abcam), anti-VAMP2 (1:1000; 104 202, SySy), anti-PSD-95 (1:1000; 124 011, SySy) and anti-tPA (2 ug/mL; ASMTPA-GF-HT, Innovative Research). Membranes were washed with 0.1% Tween-20 in 1X TBS. Membranes were incubated with 688, 700 or 800 Alexa fluor or DyLight conjugated secondary antibodies for 1 h at RT and developed using odyssey CLx imaging system (LI-COR). Images were analyzed using Image Studio Lite Ver 5.2. Membranes were cut horizontally when needed to probe for multiple proteins.

For tPA levels in whole brain of WT, tPA Ala-KI, tPA Ala-BAC, and tPA-KO mice, we used a Luminex assay. A primary antibody against mouse tPA (ASMTPA-GF-HT, Molecular Innovations) was coupled to carboxylated beads (Luminex) overnight at 4 °C. Coupled beads were then incubated for 2 h in a filter plate (MABVN0B50, Millipore) at RT in the dark with the brain protein extracts. After 3 washes with the wash buffer (PBS-0.05% Tween 20), a biotin-labeled rabbit anti-mouse tPA antibody (Molecular Innovations, ASHTPA-HT-BIO) was added for 1 h at RT. The beads were then washed 3 times with wash buffer, and streptavidin-linked R-Phycoerythrin (S866, ThermoFisher Scientific) was added for 30 min. Beads were washed again with wash buffer, resuspended in sheath fluid (Luminex), and analyzed. The Luminex 100 device was used to detect the fluorescence signal [38].

### Synaptosome preparations

Dissected SN from non-perfused WT or tPA-KO mice were manually homogenized with a glass homogenizer in synaptosome buffer (320 mM sucrose, 1mM EDTA, 5 mM Tris Base, pH 7.4) and centrifuged at 1000 × g for 5 min at 4 °C. The supernatant was collected and further centrifuged at 15,000 × g for 20 min at 4 °C to obtain the crude synaptosome preparation (pellet) and the cytosolic fraction (supernatant). The crude synaptosome preparation and cytosolic fraction were then used for Western blot analysis. A subset of crude synaptosomes from SN WT mice were used for depolarization experiments. Crude synaptosome samples were carefully resuspended and incubated for 30 min at 37 °C in artificial cerebral spinal fluid (aCSF) containing 5 mM KCl (10 mM glucose, 5 mM KCl, 140 mM NaCl, 5 mM NaHCO3, 1 mM MgCl2, 1.2 mM NaHPO4, 20 mM HEPES, 1.3 mM CaCl2) or aCSF with 50 mM KCl (10 mM glucose, 50 mM KCl, 140 mM NaCl, 5 mM NaHCO3, 1 mM MgCl2, 1.2 mM NaHPO4, 20 mM HEPES, 1.3 mM CaCl2). After incubation, crude synaptosomes were centrifuged at 15,000 × g for 15 min at 4 °C, and the free synaptosome supernatant was used for in-gel zymography analysis.

### In-gel zymography

Protein extracts from whole brain, SN, and crude synaptosome supernatant preparations were used to measure tPA proteolytical activity. Non-boiled and non-reduced protein samples were loaded in an in-house polyacrylamide gel (stacking gel: 125 mM Tris (pH 6.8), 4% acrylamide, 0.1% SDS, 0.1% APS, 0.1% TEMED; resolving gel: 375 mM Tris (pH 8.8), 10% Acrylamide, 0.1% SDS, 1 mg/mL casein, 10 ug/mL human plasminogen (HGPG-716; Molecular Innovations), 0.1% APS, 0.1% TEMED). Samples were run for 80 min at 120 V. After protein separation, gels were washed 4 times for 30 min in 2.5% Tx-100 (in water) and then briefly washed for 5 min in developing buffer containing 100 mM Tris pH 8.1. Gels were incubated in developing buffer at 37 °C for 3 h. Gels were then stained with Bio-Safe Coomassie (1610786, Bio-Rad); bands devoid of Coomassie stain (white) indicate areas of proteolytic activity [8]. tPA-KO mice extracts were used as a negative control and human tPA was used as a positive control for this assay.

### Corridor task

Lateralized sensory-motor integration was measured using a corridor task. This behavioral test was selected due to its sensitivity to detect partial unilateral DA-neuron damage in rats and mouse PD models [39, 40], which is ideal for the mild unilateral degeneration of DA-neurons observed in the SN after rAAV2-hα-SYN injection [33]. This test consists in a long narrow rectangular plexiglass corridor with the following dimensions: L = 60 cm x W = 4 cm x H = 15 cm (testing corridor). The testing corridor contains 10 pairs of adjacent Eppendorf caps placed at 5-cm intervals with 4–5 sugar pellets each (20 mg; TestDiet). A corridor without Eppendorf caps with the same dimensions as the testing corridor was used as the habituation corridor. WT and tPA-KO mice injected with rAAV2-hα-SYN or rAAV2-control virus or WT mice injected with rAAV2-hα-SYN and treated with Glunomab or vehicle intravenously were habituated 1 day before the end of the experiment (4 weeks after injection) in the habituation corridor by scattering sugar pellets along the corridor floor and allowing them to freely explore for 10 min. Lateralized sensory-motor integration was tested 4 weeks after rAAV2-hα-SYN or rAAV2-control virus injection. On the testing day, mice were placed in the habituation corridor for 5 min in the absence of sugar pellets, then mice were transferred to one end of the testing corridor containing sugar pellets and video recorded for 5 min. The video recordings were analyzed by an investigator blinded to the treatment and genotype. The number of ipsilateral and contralateral explorations relative to the injected hemisphere were counted until the mouse made a total of 20 explorations or the video ended. An exploration was defined as a nose-poke into an Eppendorf cap, whether the sugar pellet was poked or eaten, and a new exploration was only counted by exploring a new cap. Data is expressed as a percentage of bias lateralized explorations, calculated as: Bias (%) = 100*(ipsilateral - contralateral) / (ipsilateral + contralateral).

### RNA library preparation, next generation sequencing and RNA-seq analysis

RNA isolated from the dissected uninjected and injected SN of male WT and tPA-KO mice 4 weeks after rAAV2-ha-SYN injection were used for RNA library preparation and RNA-seq analysis. Following manufactured instructions, RNA was isolated using QIAzol lysis buffer and the NucleoSpin RNA kit (Cat # 740955.50; Macherey-Nagel). RNA quality control, library preparation and next-generation sequencing were performed at the advanced genomics core at the University of Michigan. Briefly, the integrity of the total isolated RNA was assessed using TapeStation RNA Tape (Agilent Technologies). RNA was quantified using Qubit RNA Assay kit (Invitrogen) and cDNA libraries were constructed using QuantSeq 3’-mRNA-Seq Library Prep Kit (Lexogen Inc., Vienna, Austria) following manufacturer instructions. High-throughput sequencing was performed on single-read 100 bp fragments using the NovaSeq 6000 (Illumina Inc). The obtained Fastq files were trimmed using Trim Galore and reads were aligned with the STAR algorithm against the mouse reference genome (ID: mm10). Read collapsing was done using Lexogen’s provided collapse UMI bam binary. To assign and count the aligned reads we used the Rsubread and Feature Counts function from Bioconductor R packages. Differential gene expression analysis was done in Bioconductor R package edgeR and Limma. The following comparisons between the injected SN hemisphere and uninjected SN hemisphere were made in WT and tPA-KO mice: Injected WT vs. Uninjected WT, Injected tPA-KO vs. Uninjected tPA-KO, Injected WT vs. Injected tPA-KO, and Uninjected WT vs. Uninjected tPA-KO. *P*-values were adjusted for multiple comparisons using false discovery rate and genes were considered significantly different when adjusted *p*-value was < 0.05 and a fold change of > 1.5 (log_2_FC of 0.58) were obtained.

Heatmap of top differentially expressed genes was generated using heatmap2 (Galaxy Project). Top differentially expressed genes of two pair-comparison were selected and combined to make the heatmap: Injected WT vs. Injected tPA-KO mice (*Wdfy1*,* Mt2*,* Nnt*,* Ctss*,* Slc7a5*,* Cpne9*,* Gabra2*,* C1qa*,* Ly6a*,* Eif5a2*) and Injected WT vs. uninjected WT mice (*Ctss*,* C1qa*,* Fcgr3*,* B2m*,* Tph2*,* Serpina3n)* (see Additional file 1). Raw counts per millions of these genes in all experimental conditions were used to generate the heatmap. Gene ontology (GO) analysis was done using the IpathwayGuide suit (ADVAITA). For this analysis, differentially expressed genes between Injected WT vs. Injected tPA-KO mice SN were selected based only on the fold change criteria (fold change of > 1.5 or log2FC of 0.58). The GO terms were adjusted for multiple comparisons using the false discovery rate method and biological process terms were selected based on the GO Consortium database (see Additional file 1).

### Quantitative RT-PCR

RNA was isolated from the dissected uninjected and injected SN of WT and tPA-KO mice 4 weeks after rAAV2-ha-SYN injection. Quantitative RT-PCR analysis was performed using SYBR Green Master Mix according to manufacture instructions (Applied Biosystems). Genes of interest were selected based on RNA-seq analysis (*C1qa*, *b2m*, *Wdfy1* and *Ctss*). Primers were generated using Primer-BLAST: *C1qa* (NM_007572.2; FWR 5’-GTT TGA TCG GAC CAC GGA GG-3’ REV 5’-AGATTCCCCTGGGTCTCCTT-3’), *b2m* (NM_009735.3; FWR 5’-CTC GGT GAC CCT GGT CTT TC-3’ REV 5’-GGA TTT CAA TGT GAG GCG GG-3’), *Ctss (NM_001267695.2;* FWR 5’-*GGA GTG AGC ACC ACA CTT CA*-3’ REV 5’*TCC CAA TGG TAG TCC AGG GT*-3’) and *wdfy-1* (NM_001111279.1; FWR 5’-GCA AGA TAG AGG GCC ACC AG-3’ REV 5’-AAA TAT CCG CCT GCT GTC GT-3’). Ct values were used to calculate fold change relative to the housekeeping gene *Rpl-38* (FWR 5’-CGC CAT GCC TCG GAA A-3’ REV 5’-CCG CCG GGC TGT CAG-3’).

### Glunomab treatment and pharmacokinetics

Both glunomab and the isotype control antibody bind to the N-terminal domain of NMDAR1. However, glunomab blocks the interaction of tPA with NMDAR1, while the isotype control antibody does not affect tPA binding to NMDAR1 [41]. Glunomab specifically binds to NMDAR1 at amino acids 176–180 [41]. Osmotic pumps (model 1004, Alzet) were filled with glunomab neutralizing antibody or Isotype control (Lys Therapeutics; France) following manufacture instructions. Osmotic pumps were connected to a brain infusion kit (Kit #3, Alzet) consisting of a catheter attached to a brain cannula. Pump assemblies were incubated for 48 h in sterile saline solution at 37 °C to ensure proper pump equilibration and drug delivery. Mice were anesthetized with 2% isoflurane and secured on a stereotactic frame. Then, an osmotic pump was implanted subcutaneously into the left lower limb of tPA Ala-BAC mice ∼ 45 min after rAAV2-hα-SYN injection into the SN. The initial skin incision and skull window used for rAAV2-hα-SYN injection in the SN were used to implant the osmotic pump subcutaneously and to place the brain cannula in the SN. The brain cannula connected to the pump was placed slightly above the SNpc to avoid physical damage of DA-neurons (AP= -3.1 ML=-1.4 DV=-3.8 mm). Glunomab or isotype control was delivered at a rate of 6ug/day for 4 weeks. Mice were singly housed for the duration of the treatment to minimize possible damage to the pump delivery system. The brain cannula position in the SN was confirmed by immunohistochemistry analysis. For intravenous glunomab treatment, male and females WT mice were injected with glunomab (10 mg/kg/weekly) or vehicle 1 h after rAAV2-hα-SYN injection into the SN. 4 weeks after treatment, mice were PBS and 4% PFA perfused, and brains were harvested for immunohistochemistry analysis.

For pharmacokinetics studies, female CD-1 mice were intravenously injected with ^125^ISIB-glunomab at 10 mg/kg. Plasma and whole blood were collected at different timepoints (5 min, 1, 4, 6, 24, 48, 72, 96, 120, 168, 336, 504 and 672 h), and glunomab radioactivity was measured. Mean terminal half-life and mean residence time were calculated.

### Statistical analysis

Data analysis was performed using GraphPad Prism 8 statistical software (GraphPad Software, La Jolla, CA, USA). All experiments were repeated at least two independent times and n indicates the number of individual mice used in the study. For statistical analysis, in any experiment with only two groups, a two-tailed t-test was used. For experiments with more than two groups, a one-way ANOVA with Tukey post hoc test was used. Normal distribution and homogeneity of variance was confirmed before using t-test or ANOVA. Data is represented as mean values ± S.E.M; *p* < 0.05 was considered significant.

## Results

### tPA is localized in presynaptic terminals in the SN

**Fig. 1:**
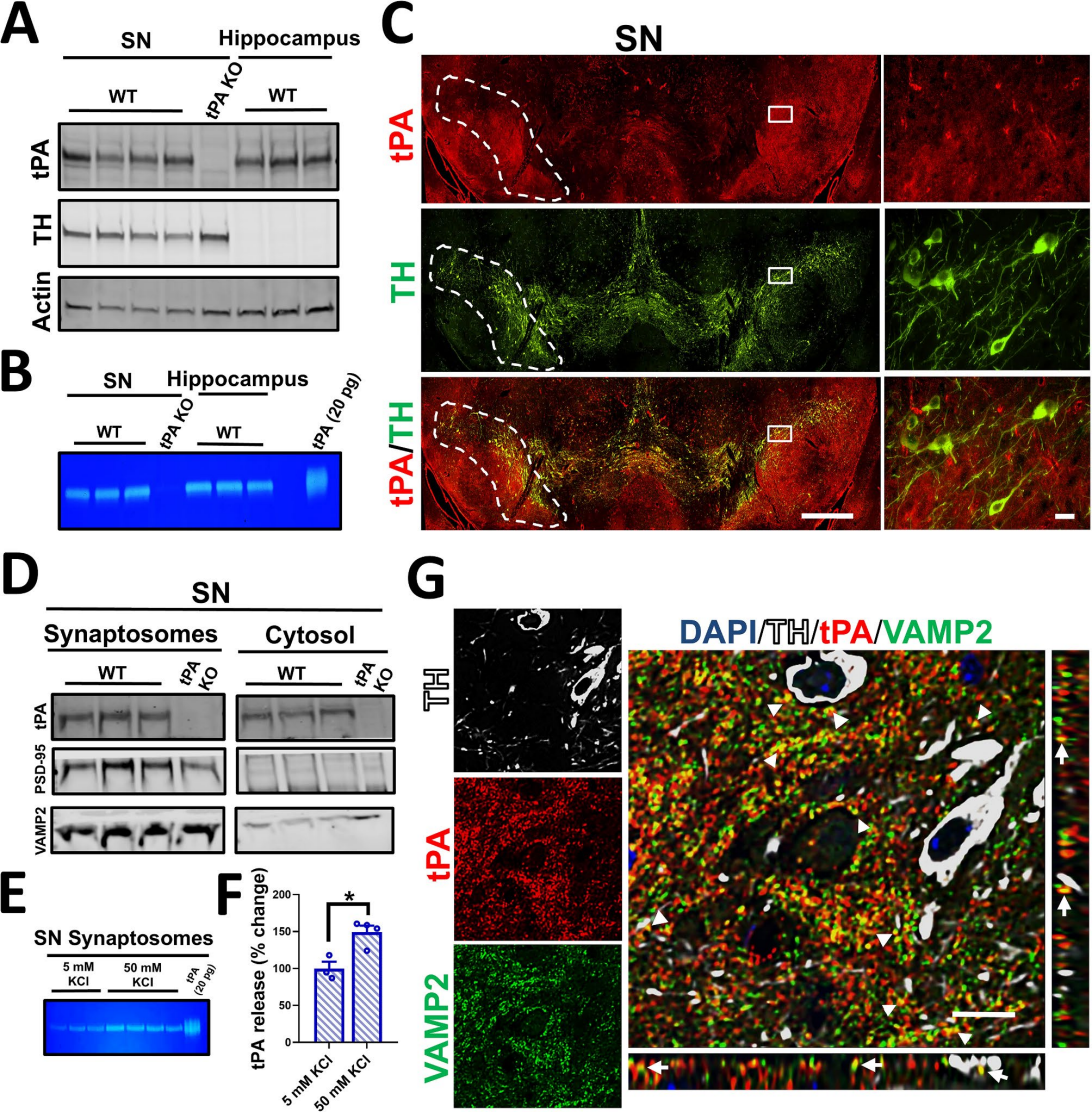
tPA is localized to presynaptic terminals associated with DA-neurons. (**A**) Western blot of tPA, TH, and actin in the SN and hippocampus of WT mice (*n* = 3–4). Actin was used as a loading control. (**B**) In-gel zymography showing tPA proteolytic activity in the SN and hippocampus of WT mice. Human tPA was used as a positive control (*n* = 3). (**C**) Coronal sections of the SN showing tPA (red) and TH (green) staining in WT mice. White squares indicate 60x closeup (right panels) and white dashed lines depict SNpc (*n* = 3). (**D**) Western blot of crude synaptosome protein extracts from SN of WT mice. Synaptosome and cytosol fractions were run and developed in the same gel and membrane (*n* = 3). (**E**) In-gel zymography showing tPA proteolytic activity, and (**F**) quantification of band intensity of supernatants from crude synaptosome preparations from the SN after 5 mM or 50 mM KCl treatment (*n* = 3–4). tPA-KO mice were used as a negative control in (**A**), (**B**), and (**D**). (**G**) Confocal images of the SN showing tPA (red), TH (white), and VAMP-2 (green). Merged image shows colocalization of tPA and VAMP-2 (yellow and white arrows). Panels next to merged image (xy) are orthogonal view indicating xz (bottom panel) and yz (right panel) colocalization (*n* = 3; MOC = 0.4 ± 0.04). Data are shown as mean ± SEM, *p < 0.05; two-tailed t-test. Scale bar = 500 μm (**C**), 20 μm (close up, **C**), 10 μm (**G**)

In previous studies using tPA reporter mouse lines, we found tPA protein in the SN yet minimal tPA gene expression [8]. However, the detailed characterization of tPA expression and its role in SN physiology and pathology remained unexplored. To confirm tPA localization under normal conditions, SN from naïve WT mice were dissected and tPA levels determined by western blot. We found high levels of tPA protein in the SN that were similar to those found in the hippocampus, a structure known to have high tPA expression and activity [8, 9, 17]. Mice lacking tPA (tPA-KO) served as a negative control, and tyrosine hydroxylase (TH), a marker for DA-neurons, was used to verify the SN dissection (Fig. 1A). In-gel plasminogen-dependent zymography confirmed that tPA in the SN was proteolytically active (Fig. 1B). The SN can be divided into two morphologically distinct regions: SNpc, containing DA-neurons, and SN *pars reticulata*, containing GABAergic neurons. To test if tPA was associated with DA-neurons, we stained coronal brain sections of the WT mouse SN for tPA and TH. We found that tPA was located proximal to DA-neuron (TH^+^) cell bodies and axons but was not expressed by these neurons in the SN (Fig. 1C).

In the brain, tPA is trafficked and released by axons of cortical and hippocampal neurons [8, 16, 42]. To explore if tPA could be associated with axon terminals in the SN, we extracted crude synaptosomes from the SN of naïve WT mice and measured tPA levels. We found that tPA was enriched in crude synaptosome preparations, suggesting that tPA could be associated with synaptic terminals in the SN (Fig. 1D). tPA was also found in the cytosolic fraction of the SN extracts. Cytosolic tPA could be explained by tPA expression in cerebral blood vessels [25]. The presynaptic vesicle-associated membrane protein 2 (VAMP-2) and postsynaptic density protein 95 (PSD-95) were used as controls for synaptosome enrichment (Fig. 1D). To test whether tPA could be released from synaptic terminals after membrane depolarization, crude synaptosomes obtained from the SN were exposed to 5 or 50 mM KCl for 30 min. There was a significant increase in tPA activity in synaptosome supernatants treated with 50 mM KCl compared to those treated with 5 mM KCl (Fig. 1E, F). In primary cortical neurons tPA colocalizes with pre- and postsynaptic vesicles [42]. To determine if tPA expressed in the SN colocalizes with presynaptic axons in contact with DA-neurons, the SN was stained for the presynaptic vesicle marker VAMP-2 (green), tPA (red), and TH (white) in WT mice. Confocal microscopy showed that tPA colocalizes with VAMP2 proximal to TH^+^ axons in the SN (Fig. 1G). Quantification of the colocalization between tPA and VAMP-2 was performed using Mander’s overlap coefficient (MOC = 0.4 ± 0.04; *n* = 3). Altogether, these data suggest that tPA is present in presynaptic terminals proximal to DA-neurons in the SN.

### DARPP-32 GABAergic neurons in the striatum send tPA^+^projections to the SN

**Fig. 2:**
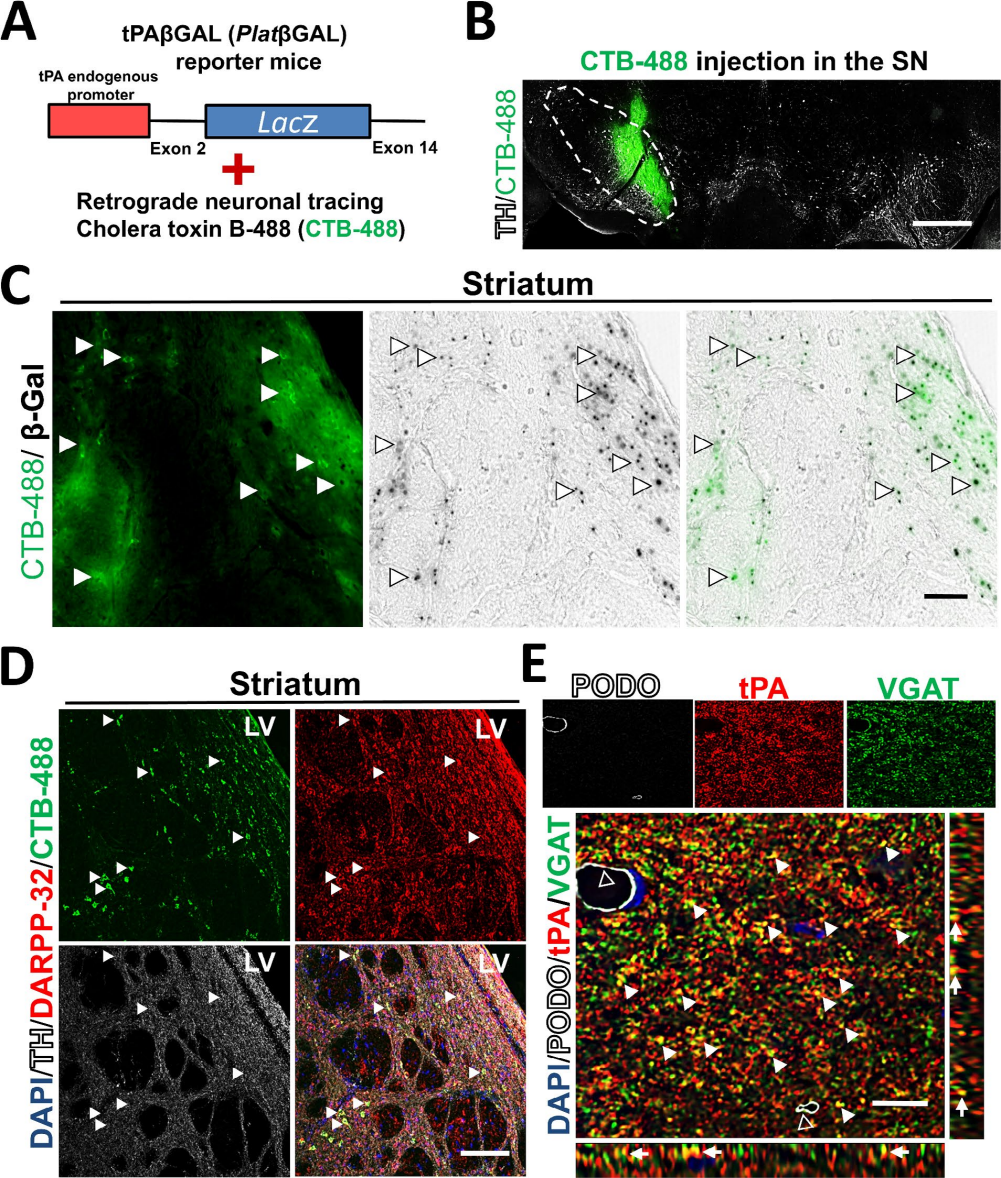
DARPP-32 GABAergic neurons in the striatum send tPA^+^projections to the SN. (**A**, **B**) Retrograde tracing strategy using tPAβGAL reporter mice showing CTB-488 injection site (green) in the SN. (**C**) Representative coronal section images of the dorsomedial striatum showing β-Gal^+^ cells (black) and CTB-488^+^ neurons (green) in tPAβGAL^+/−^ mice 1 week after CTB-488 injection in the SN. White arrows indicate double-positive CTB-488 and β-Gal cells. Only the striatum showed CTB-488^+^ staining in neurons (average number of CTB-488^+^ in striatum per mm^2^ = 272.3 ± 41.6; *n* = 3; green). (**D**) Coronal sections of the striatum showing DARPP-32 (red), TH (white), CTB-488 (green), and DAPI (blue) staining in tPAβGAL^+/−^ mice 1 week after CTB-488 injection in SN. White arrows indicate double-positive CTB-488 and DARPP-32 cells in the dorsomedial striatum (*n* = 4). (**E**) Confocal images of the SN showing tPA (red), the blood vessel marker podocalyxin (PODO; white) and VGAT (green). PODO staining was used to show that tPA in the SN is mainly associated with GABAergic presynaptic axons and not blood vessel (white open arrows). Merged image (xy) shows colocalization of tPA and VGAT (yellow and white arrows). Panels next to merged image (xy) show orthogonal view indicating xz (bottom panel) and yz (right panel) colocalization (*n* = 3; MOC = 0.39 ± 0.05). Scale bar= (**B**) 500 μm (**C**) 50 μm (**D**) 100 μm (**E**) 10 μm

tPA colocalizes with presynaptic terminals (Fig. 1D, G); however, the origin and nature of these tPA^+^ neurons projecting to the SN are unknown. To identify the subpopulation(s) of neurons sending tPA^+^ projections to the SN, we used the axonal retrograde tracer cholera toxin B-488 (CTB-488) in combination with tPA reporter mice (PlatβGAL) [8]. In these mice, β-galactosidase (βGAL) expression is driven by the endogenous tPA promoter (Fig. 2A). These reporter mice identify tPA-expressing cell bodies (βGAL^+^ cells) but do not show βGAL protein trafficking [8]. Neurons positive for both CTB-488 and βGAL after intranigral CTB-488 delivery in the PlatβGAL mice (Fig. 2B) will identify candidate tPA-expressing neurons that project to the SN. We found that the only CTB-488^+^/βGAL^+^ population of neuronal cell bodies was in the striatum, proximal to the lateral ventricles in the dorsal region of the striatum (Fig. 2C and Fig. S2). These data indicate that a subpopulation of striatal neurons expressing tPA send projections to the SN.

Approximately 90% of neurons in the striatum are GABAergic and send projections to different brain regions, including the SN [43]. These striatal GABAergic neurons express dopamine- and cAMP-regulated neuronal phosphoprotein of molecular weight 32 kDa (DARPP-32) [44]. To determine if CTB-488^+^ striatal neurons are GABAergic neurons, we stained the striatum with DARPP-32 in *Plat*βGAL mice after CTB-488 injection in the SN. We found that most CTB-488^+^ neurons in the striatum were DARPP-32^+^ (∼ 70% ± 0.04; *n* = 3) and were contacted by TH^+^ axons (Fig. 2D). Consistent with these results, we found that in the SN, tPA colocalizes with the GABAergic presynaptic marker vesicular GABA transporter (VGAT; Fig. 2E). No significant colocalization was found in the SN between tPA and two glutamatergic presynaptic markers, the vesicular glutamate transporter (VGLUT) 1 or 2 (Fig. S3A) [7]. Colocalization between tPA and VGAT was confirmed using MOC (MOC = 0.39 ± 0.05; *n* = 3). Overall, these data suggest a potential unrecognized role for tPA in the interaction between striatal GABAergic and nigral DA-neurons, which could have clinical relevance in DA-neuronal degeneration.

### tPA-KO mice are protected from α-synuclein overexpression-induced DA-neuron degeneration and behavioral deficits

**Fig. 3:**
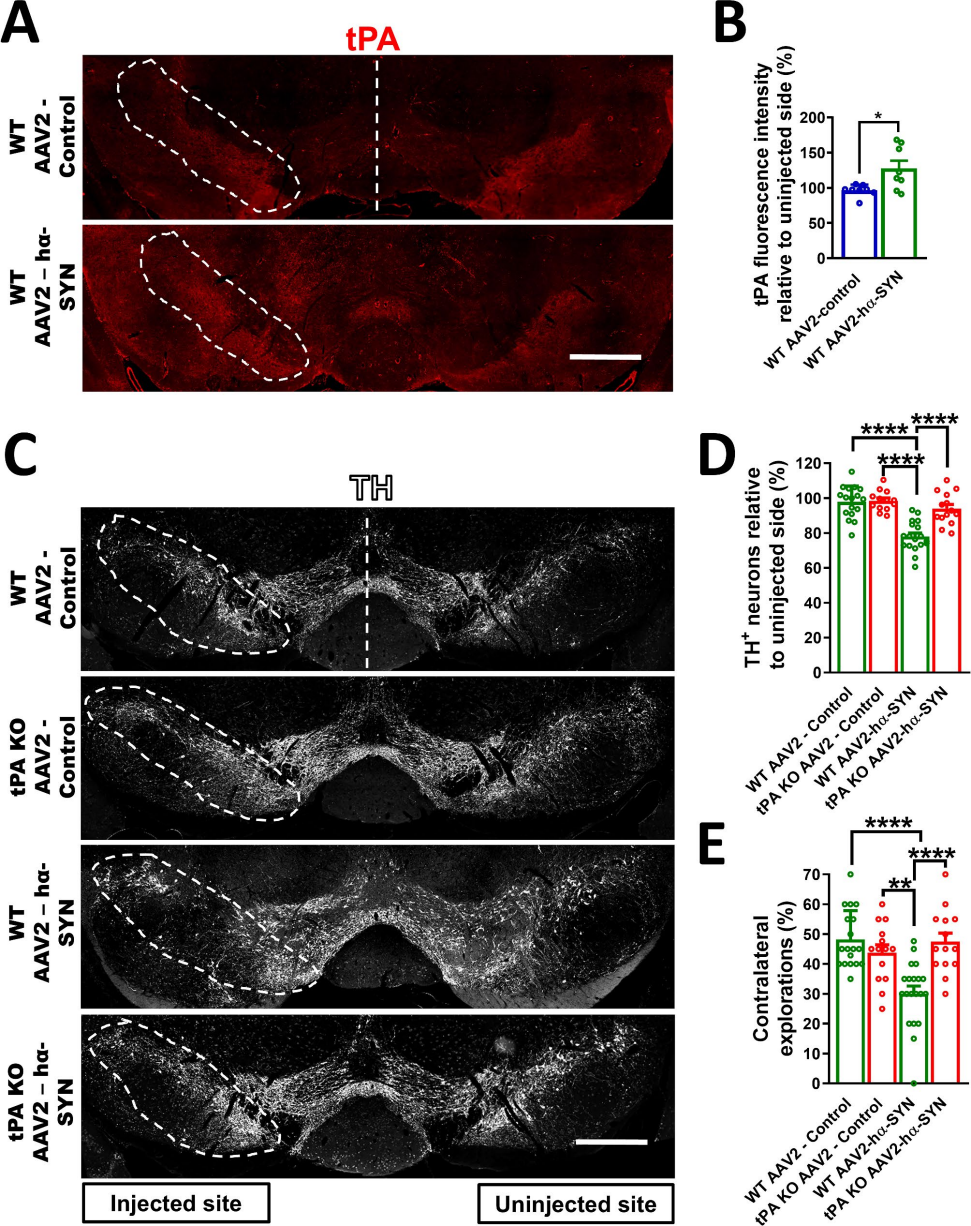
tPA-KO mice are protected from DA-neuron degeneration and behavioral deficits after rAAV2-hα-SYN injection. (**A**) Coronal sections of the SN showing tPA staining and (**B**) quantification of tPA fluorescence intensity in the SN 4 weeks after rAAV2-hα-SYN or rAAV2-control injection in WT mice (*n* = 8–9). (**C**) Representative images of the SN showing DA-neuron degeneration in the injected SN (TH, white), and (**D**) quantification of TH^+^ neurons in coronal sections of the SN 4 weeks after rAAV2-hα-SYN or rAAV2-control injection in WT and tPA-KO mice. (**E**) Quantification of sensorimotor bias in a corridor task 4 weeks after rAAV2-hα-SYN or rAAV2-control injection in WT and tPA-KO mice. White dashed lines represent the SNpc (*n* = 14–20). These experiments were conducted using 9- to 11-week-old WT and tPA KO mice. Data are shown as mean ± SEM, N.S = not significant, *p < 0.05, **p < 0.01; ****p < 0.0001. (**B**) 2-tailed t-test, (**D**,** E**) 1-way ANOVA followed by Tukey post hoc test. Scale bar = 500 μm

Based on tPA’s potential for neurotoxicity in the CNS [24–26], we explored whether tPA expression could affect DA-neuron survival in the SN. We used a mouse model of PD where overexpression of human α-synuclein (hα-SYN) in DA-neurons is driven by the rAAV2 virus (rAAV2-hα-SYN) [45]. Both WT and tPA-KO mice were unilaterally injected in the SN with either an empty rAAV2 virus (control) or a rAAV2-hα-SYN virus. DA-neuron degeneration was quantified in the SN 4 weeks after virus injection. This time point was selected based on our previous research showing that this early stage is sufficient to observe significant neuroinflammation and neurodegeneration in this PD mouse model [33]. We found that in WT mice, overexpression of hα-SYN significantly increased tPA immunoreactivity in the SN compared to control virus-injected WT mice, suggesting that tPA was increased in response to overexpression of hα-SYN (Fig. 3A, B). As expected, there was a significant loss of TH^+^ neurons in WT mice injected with the rAAV2-hα-SYN compared to WT mice injected with control virus (Fig. 3A, B). In contrast, mice lacking tPA showed significant protection of DA-neurons compared to WT mice after rAAV2-hα-SYN injection (Fig. 3C, D). DA-neuron degeneration was not observed with the empty control virus in either WT or tPA-KO mice (Fig. 3C, D), indicating that neuronal loss was due to overexpression of hα-SYN. Similar levels of hα-SYN expression were confirmed in the SN of both WT and tPA-KO mice (Fig. S3C, D).

To assess whether the lack of tPA prevents the development of behavioral deficits after rAAV2-hα-SYN injection, a lateralized sensory-motor integration test was used. In this behavioral test, contralateral and ipsilateral explorations of sugar pellets in an uninjured mouse are expected to have the same probability (∼ 50% contralateral or ipsilateral explorations; no bias) [33, 39, 40]. We observed a significant reduction in contralateral explorations 4 weeks after rAAV2-hα-SYN injection in WT mice compared to control virus-injected WT mice. However, consistent with the significant protection of DA-neurons, tPA-KO mice showed no bias in explorations after rAAV2-hα-SYN injection (Fig. 3E).

### Genes associated with innate immune responses are upregulated by overexpression of hα-SYN in WT mice, but not in tPA-KO mice

**Fig. 4:**
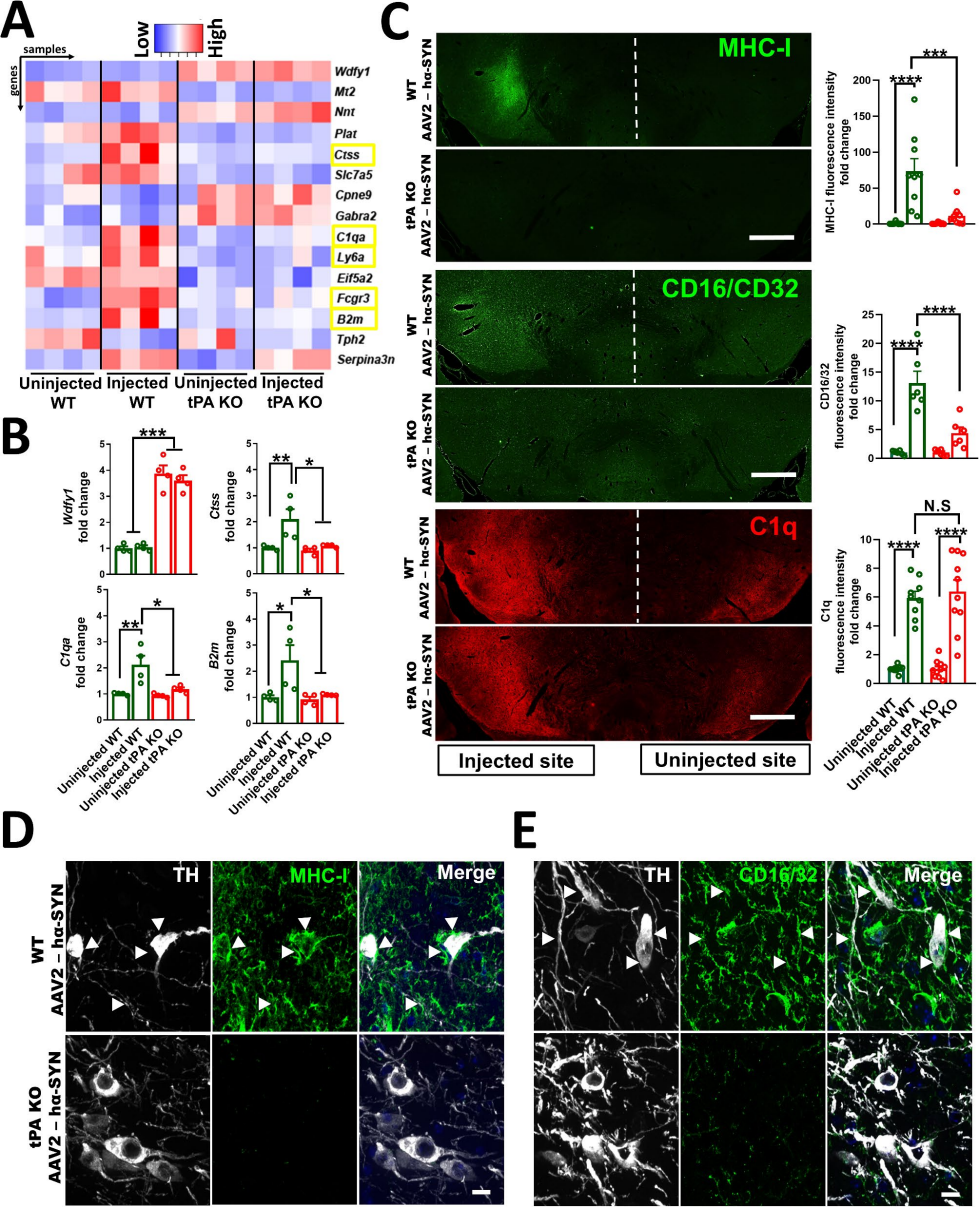
Genes and proteins associated with innate immune response are upregulated by overexpression of hα-SYN in the SN of WT mice, but not in tPA-KO mice. (**A**) Heatmap showing combined differentially expressed genes in the SN between uninjected and injected SN for WT and tPA-KO mice 4 weeks after rAAV2-hα-SYN injection in the SN. Yellow rectangles indicate genes associated with innate and adaptive immune response downregulated in tPA-KO mice compared to WT mice 4 weeks after rAAV2-hα-SYN (*n* = 4). Heatmap was generated using the raw counts per millions of the selected genes. (**B**) q-PCR validation of selected genes of interest in the SN 4 weeks after rAAV2-hα-SYN injection in the SN. *RLP38* was used as the housekeeping gene (*n* = 4). (**C**) Coronal sections of the SN showing MHC-I, CD16/32, and C1q staining and their respective quantifications indicating fluorescence intensity fold change relative to the uninjected site 4 weeks after rAAV2-hα-SYN injection in WT and tPA-KO mice (*n* = 6–10). Confocal images of the SN showing TH (white), (**D**) MHC-I, or (**E**) CD16/32 (green) staining in WT and tPA-KO mice 4 weeks after rAAV2-hα-SYN injection (*n* = 3–5). Arrows indicate MHC-I or CD16/32 in contact with TH^+^ cell bodies or axons in WT mice after rAAV2-hα-SYN injection. Confocal images are shown as maximum intensity projections. For (A) and (B) experiments were conducted using 12- to 13-week-old WT and tPA KO mice. For (C-E) experiments were conducted using 9- to 11-week-old WT and tPA KO mice. Data are shown as mean ± SEM, N.S = not significant, *p < 0.05 **p < 0.01; ***p < 0.001; ****p < 0.0001. (a-c) 1-way ANOVA followed by Tukey post hoc test. Scale bar= (**C**) 500 μm **(D**,** E**) 10 μm

To explore the possible mechanism(s) driving DA-neuron degeneration in the context of hα-SYN neurotoxicity, we analyzed differential gene expression in the SN via RNA-seq in WT and tPA-KO mice 4 weeks after rAAV2-hα-SYN injection. We observed that compared to the uninjected hemisphere, the overexpression of hα-Syn in WT mice significantly upregulated the expression of genes involved in antigen processing and presentation and the innate immune response, including *b2m*, *Ctss*, *FcgRIII*,* Ly6A*, and *C1qa*. In contrast, overexpression of hα-Syn in tPA-KO mice did not significantly increase the expression of these genes compared to either WT or tPA-KO uninjected SN (Fig. 4A, Fig. S4A, and Additional file 1). These results were confirmed by quantitative PCR in genes of interest (Fig. 4B). The top differentially upregulated gene involved in innate immune response between WT and tPA-KO mice was *wdfy1* (Fig. 4A, B). This gene has been recently linked with inflammation and neuronal differentiation in the brain [46, 47]. However, we found that tPA/WDFY-1 double-KO mice showed protection of DA-neurons compared to WDFY-1-KO mice after rAAV2-hα-SYN injection, suggesting that baseline upregulation of WDFY-1 in tPA-KO mice was not a significant driver of neuroprotection (Fig. S4B, C).

We next focused on genes involved in inflammation that were only upregulated in WT mice injected with rAAV2-hα-SYN but not in tPA-KO mice, such as *b2m*, *C1qa*, and *FcgRIII*. These genes are associated with inflammation and neurodegeneration in PD pathology [48–50]. To confirm if the differential gene expression data were in agreement with protein levels in the SN, we stained the SN of WT and tPA-KO mice 4 weeks after rAAV2-hα-SYN injection for complement-1 q (C1q), major histocompatibility complex class I (MHC-I; *b2m* encodes a subunit of this receptor), and CD16/CD32 (*FcgRIII* encodes the CD16 subunit of this heteroreceptor). MHC-I and CD16/CD32 proteins were significantly reduced in the SN of tPA-KO mice compared to WT mice after rAAV2-hα-SYN injection. Interestingly, C1q immunoreactivity was significantly increased in tPA-KO mice after rAAV2-hα-SYN injection compared to uninjected control SN in WT and tPA-KO mice (Fig. 4C), suggesting that C1q might not be involved in tPA-mediated DA-neuron degeneration. Confocal images showed MHC-I and CD16/CD32 staining in close contact with DA-neuron cell bodies and axons (TH; white) in the SN of WT mice after overexpression of hα-SYN. In contrast, MHC-I and CD16/CD32 were reduced and not associated with DA-neurons in tPA-KO mice after overexpression of hα-SYN (Fig. 4D, E). Injection of rAAV2-control in WT mice did not show a significant increase in MHC-I or CD16/32 expression in the injected SN compared to the uninjected control. However, C1q showed a non-significant increase, suggesting that the C1q increase may be related to the mechanical injury from the injection and independent of hα-SYN overexpression in the SN (Fig. S4D).

### tPA deficiency reduces proinflammatory microglia and infiltrated T-cells in the SN after overexpression of hα-SYN

**Fig. 5:**
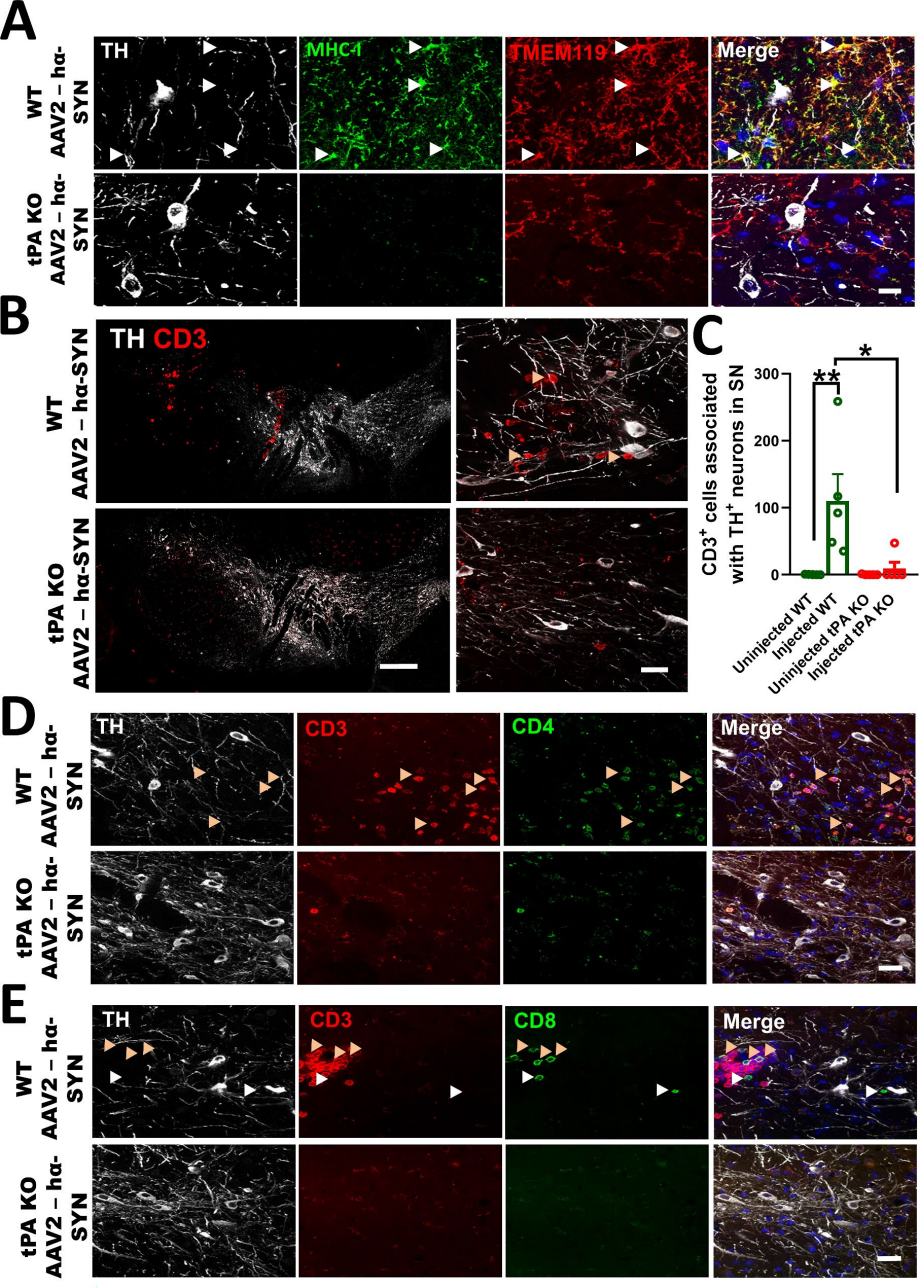
Lack of tPA reduced proinflammatory microglia and infiltrated T-cells in the SN after overexpression of hα-SYN. (**A**) Confocal images of the SN showing TMEM119 (red), TH (white), and MHC-I (green) staining 4 weeks after rAAV2-hα-SYN injection in WT and tPA-KO mice. Arrows indicate MHC-I colocalization with TMEM119 (MOC = 0.8 ± 0.1; *n* = 3). (**B**) Representative images of the SN showing CD3 (red) and TH (white) staining with closeup showing CD3^+^ cells in contact with TH cell bodies and axons (arrows). (**C**) Quantification of CD3^+^ T cells associated with the SNpc 4 weeks after rAAV2-hα-SYN in WT and tPA-KO mice (*n* = 5). (**D**) Confocal images of the SN showing CD3 (red), TH (white), and CD4 (green) staining or (**E**) CD3 (red), TH (white), and CD8 (green) staining in WT and tPA-KO mice 4 weeks after rAAV2-hα-SYN injection (*n* = 5). Orange arrows show the presence of CD4^+^ or CD8^+^ T cells associated with TH^+^ cell bodies and axons. (**E**) White arrows show the presence of CD8^+^/CD3^−^ cells. Confocal images are shown as maximum intensity projections. These experiments were conducted using 9- to 11-week-old WT and tPA KO mice. Data are shown as mean ± SEM, N.S = not significant, *p < 0.05; **p < 0.01; 1-way ANOVA followed by Tukey post hoc test. Scale bar= (**A**) 10 μm (**B**) 250 μm (**B** close up) 20 μm **(D**,** E**) 20 μm

CD16/CD32 is a proinflammatory marker in microglia [51, 52]. MHC-I is also associated with inflammation in the CNS and is reported to be expressed in multiple cell types, including microglia, neurons, and endothelial cells. Increased MHC-I expression has also been associated with PD pathology [53–55]. To characterize the cell types expressing CD16/CD32 and MHC-I in the SN after overexpression of hα-SYN, we performed confocal microscopy in the SN with microglial marker TMEM119, TH, and CD16/CD32 or MHC-I in WT and tPA-KO mice 4 weeks after rAAV2-hα-SYN injection. In WT mice overexpressing hα-SYN, CD16/CD32 and MHC-I mostly colocalized with TMEM119. These cells were in close contact with TH^+^ axons and cell bodies but were not expressed by DA-neurons (Fig. 5A and Fig. S5A). MHC-I and CD16/32 staining was also found in cells close to blood vessels negative for TMEM119, which could be indicative of infiltrating cells. Additionally, MHC-I colocalized with blood vessels in WT mice after overexpressing hα-SYN (Fig. S5B, C). In contrast, MHC-I and CD16/CD32 staining was reduced in tPA-KO mice in microglia close to DA-neurons after overexpression of hα-SYN (Fig. 5A and Fig. S5A). These data suggest that tPA is needed to enhance microglial activation and for the infiltration of immune cells in the context of hα-SYN-mediated damage.

Previous reports link T-cell mediated neurotoxicity with DA-neuron degeneration in both animal models and PD patients [56]. We found that three genes associated with T-cell responses, *ctss*,* b2m*,* and Ly6a*, were upregulated in the SN of WT mice compared to tPA-KO mice after rAAV2-hα-SYN injection (Fig. 4A, B). *b2m* is a necessary subunit for MHC-I, and *ctss* expression has been linked with the processing of MHC class-II peptides [57]. These two proteins are important for antigen presentation and activation of CD8 and CD4 T-cells, respectively. In addition, Ly6a upregulation has been linked with T-cell activation [58, 59]. To test if the lack of tPA affected infiltration of T-cells in the SN after overexpression of hα-SYN, we stained WT and tPA-KO mice 4 weeks after rAAV2-hα-SYN injection for T-cell markers CD3, CD8, and CD4. We found that there was a significant reduction of CD3^+^ cells associated with DA-neurons in the SN in tPA-KO mice compared to WT mice after overexpression of hα-SYN (Fig. 5B, C). In WT mice the majority of CD3^+^ cells were CD4^+^ T-cells (∼ 80%±1.2 of the CD3^+^ cells in the SN were CD4^+^; *n* = 5). CD3^+^/CD8^+^ T-cells were also detected in the SN of WT mice and accounted for ∼ 14%±1.3 of all CD3^+^ cells in the SN (*n* = 5; Fig. 5D, E). A small population of cells was also found to be CD3^−^/CD8^+^ (Fig. 5E; white arrows), possibly CD8^+^ monocytes or NK cells, as both have been linked to neurodegeneration in multiple sclerosis [60, 61]. Together, these data support the idea that tPA is necessary for the proper activation and recruitment of innate and adaptive immune cells in hα-SYN-mediated neurotoxicity in the SN.

### tPA-mediated DA-neuron degeneration is independent of its proteolytic activity in the SN

**Fig. 6:**
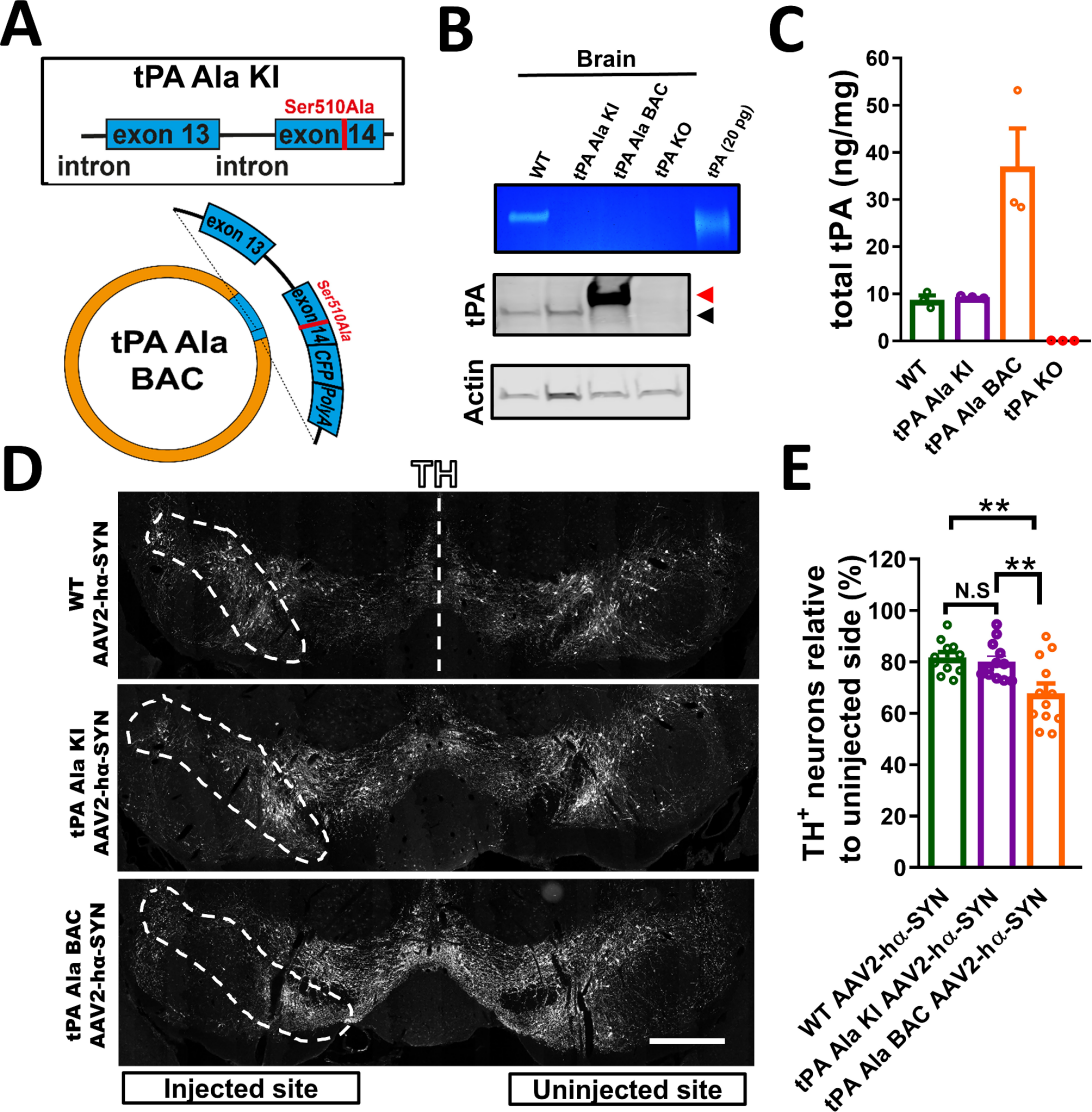
tPA-mediated DA-neuron degeneration is independent of its proteolytic activity in the SN. (**A**) Diagram depicting point mutation in exon 14 in tPA Ala-KI mice (top) and tPA Ala-BAC construct used to generate tPA Ala-BAC transgenic mice (bottom). (**B**) In-gel zymography (top; blue and white) and western blot (bottom; black and white) of whole-brain protein extract from WT, tPA Ala-KI, tPA Ala-BAC, and tPA-KO mice showing lack of tPA activity in tPA Ala-KI and tPA Ala-BAC mice but the presence of tPA protein in these mice via western blot (black arrow indicates endogenous tPA and tPA Ala; red arrow indicate the size shift in tPA Ala-Cerulean in the tPA Ala-BAC mice). (**C**) Luminex assay showing tPA levels in whole brain protein extracts in WT, tPA Ala-KI, tPA Ala-BAC, and tPA-KO mice (*n* = 3). tPA-KO mouse brain protein extracts were used as a negative control for in-gel zymography, western blot, and Luminex assay (b-c). (**D**) Representative images of the SN showing DA-neuron degeneration in the injected SN (TH; white), and (**E**) quantification of TH^+^ neurons in coronal sections of the SN 4 weeks after rAAV2-hα-SYN in WT, tPA Ala-KI, and tPA Ala-BAC mice. These experiments were conducted using 15-week-old WT mice, 13- to 14-week-old tPA Ala-KI mice, and 12- to 18-week-old tPA Ala-BAC mice. White dashed lines represent the SNpc (*n* = 11–12). Data are shown as mean ± SEM, N.S = not significant, **p < 0.01. 1-way ANOVA followed by Tukey post hoc test. Scale bar = 500 μm

tPA proteolytic activity is primarily regulated by its two main inhibitors: neuroserpin (Nsp) and plasminogen activator inhibitor-1 (PAI-1). In mouse models of ischemic stroke or seizures, Nsp deficiency (Nsp-KO) increases neuronal cell death and worsens outcomes, which is attributed to an increase in tPA proteolytic activity in the brain [25, 38]. Compared to WT mice, Nsp-KO or double Nsp and PAI-1 (Nsp/PAI-1-KO) mice did not show increased degeneration of DA-neurons after hα-Syn overexpression (Fig. S6A-C). This result suggests that the two main inhibitors of tPA are not involved in tPA-mediated degeneration of DA-neurons and that tPA-mediated neurotoxicity in the hα-Syn model of PD could be independent of tPA’s proteolytic activity. To test this hypothesis, we developed two novel transgenic mice: tPA Ala-KI and tPA Ala-BAC mice. The tPA Ala-KI mice were generated using CRISPR to “knock-in” (KI) a point mutation in the tPA locus converting Ser 510 in the tPA active site to Ala. This results in expression of a proteolytically inactive tPA (Fig. 6A-C). The tPA Ala-BAC mice were generated using a bacterial artificial chromosome (BAC) as previously described for WT tPA [8] and crossed into tPA-KO mice. They express the same proteolytically inactive tPA mutant (Ser510Ala) fused with cerulean fluorescent protein, and overexpress tPA from the endogenous tPA promoter due to insertion of multiple copies of the tPA Ala-BAC construct (Fig. 6A-C). We found that overexpression of hα-SYN in tPA Ala-KI mice was sufficient to induce DA-neuron degeneration identical to WT mice 4 weeks after rAAV2-hα-SYN injection. In contrast, increased expression of proteolytically inactive tPA in the tPA Ala-BAC mice resulted in greater DA-neuron degeneration compared to WT and tPA Ala-KI mice after rAAV2-hα-SYN injection, suggesting a dose response for the action of tPA (Fig. 6D, E). The expression of hα-SYN in DA-neurons was confirmed in all three genotypes (Fig. S6D, E). Consistent with the increase in DA-neuron degeneration, we also found that overexpression of proteolytically inactive tPA significantly increased MHC-I immunoreactivity and CD3^+^ T-cells in the SN compared to WT and tPA Ala-KI mice after rAAV2-hα-SYN injection (Fig. S7A-D). Notably, the different genotypes did not exhibit any baseline differences in the total number of TH + neurons in the uninjected SN (Fig. S7E). These data demonstrate that tPA proteolytic activity is not necessary for the degeneration of DA-neurons in the SN and points to a proteolysis-independent mechanism of tPA neurotoxicity involving the activation and recruitment of innate and adaptive immune cells.

### Interaction of tPA with NMDAR1 is necessary for inducing DA-neuron degeneration

**Fig. 7:**
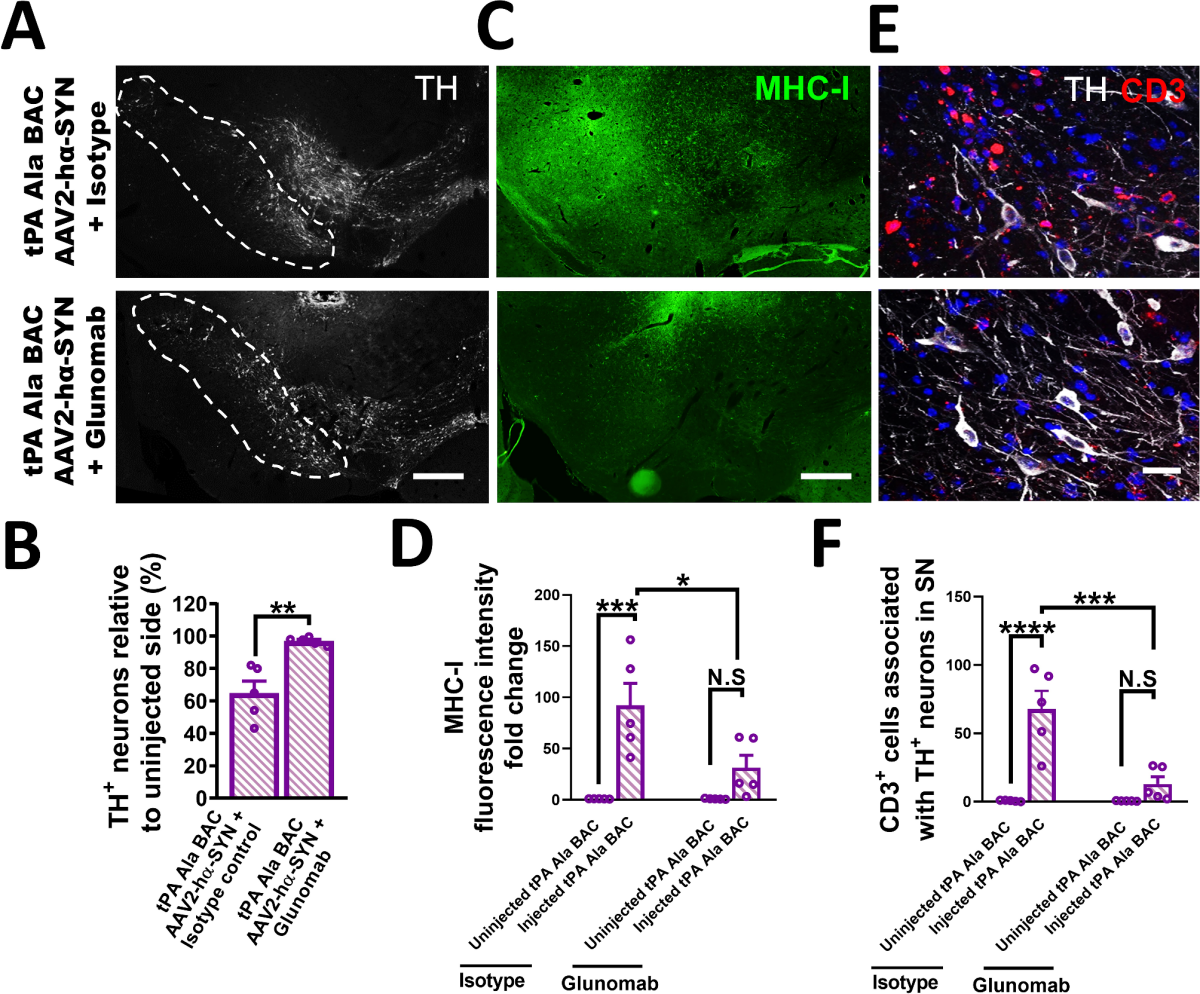
tPA-NMDAR1 interaction is necessary to induce DA-neuron degeneration in the SN. Representative images of the SN staining with (**A**) TH and (**C**) MHC-I, and the respective quantification of (**B**) DA-neuron survival and (**D**) MHC-I fluorescence intensity fold change in the SN 4 weeks after rAAV2-hα-SYN injection in tPA Ala-BAC mice treated with glunomab or isotype control (6 ug/day) (*n* = 5). (**E**) Confocal image of the SN showing CD3^+^ cells (red) in contact with TH^+^ cell bodies and axons. (**F**) Quantification of CD3^+^ T cells associated with the SNpc 4 weeks after rAAV2-hα-SYN in tPA Ala-BAC mice treated with glunomab or isotype control (*n* = 5). These experiments were conducted using 14- to 16-week-old tPA Ala-BAC mice. Data are shown as mean ± SEM, N.S = not significant, *p < 0.05; **p < 0.01; ***p < 0.001; ****p < 0.0001. (**B**) 2-tailed t-test; **(D**,** F**)1-way ANOVA followed by Tukey post hoc test. Scale bar= **(A**,** C**) 250 μm, (**E**) 20 μm

Several studies have shown that the interaction of tPA with the NMDAR subunit 1 (NMDAR1) is associated with neuronal cell death and inflammation [28, 29, 41], making the NMDAR a potential downstream candidate of tPA-mediated DA-neuron degeneration and inflammation. NMDAR1 is expressed by DA-neurons in the SN (Fig. S8A). To confirm if a non-proteolytic interaction of tPA with the NMDAR1 is involved in DA-neuron degeneration, we used glunomab, an antibody directed against the N-terminus of NMDAR1 that blocks tPA from interacting with NMDAR1 without affecting basal NMDAR1 signaling [41]. An isotype antibody that also binds to the N-terminal domain of NMDAR1 without affecting tPA-NMDAR1 binding was used as a control (isotype control) [41]. Glunomab or isotype control antibody was delivered directly to the SN via an osmotic pump connected to a brain cannula in mice that overexpress proteolytically inactive tPA (tPA Ala-BAC mice) after rAAV2-hα-SYN injection (6ug/day; Fig. S8B). Glunomab treatment showed significant protection of DA-neurons in the SN compared to isotype control-treated tPA Ala-BAC mice 4 weeks after rAAV2-hα-SYN (Fig. 7A, B). MHC-I levels and CD3^+^ T-cells infiltration were also significantly reduced in the SN after glunomab treatment compared to control mice (Fig. 7C-F). No significant reduction of CD16/CD32 or C1q levels in the SN were observed after glunomab treatment (Fig. S8C, D). The expression of hα-SYN in DA-neurons was confirmed in glunomab- and isotype control-treated tPA Ala-BAC mice (Fig. S8E, F). Overall, these data indicates that tPA promotes DA-neuron degeneration independent of its proteolytic activity. Instead, the tPA-NMDAR1 interaction increases neuroinflammation by intensifying microglial activation and lymphocyte infiltration.

### Systemic delivery of glunomab reduces neuroinflammation and DA-neuron degeneration and rescues behavioral deficits after hα-SYN overexpression

**Fig. 8:**
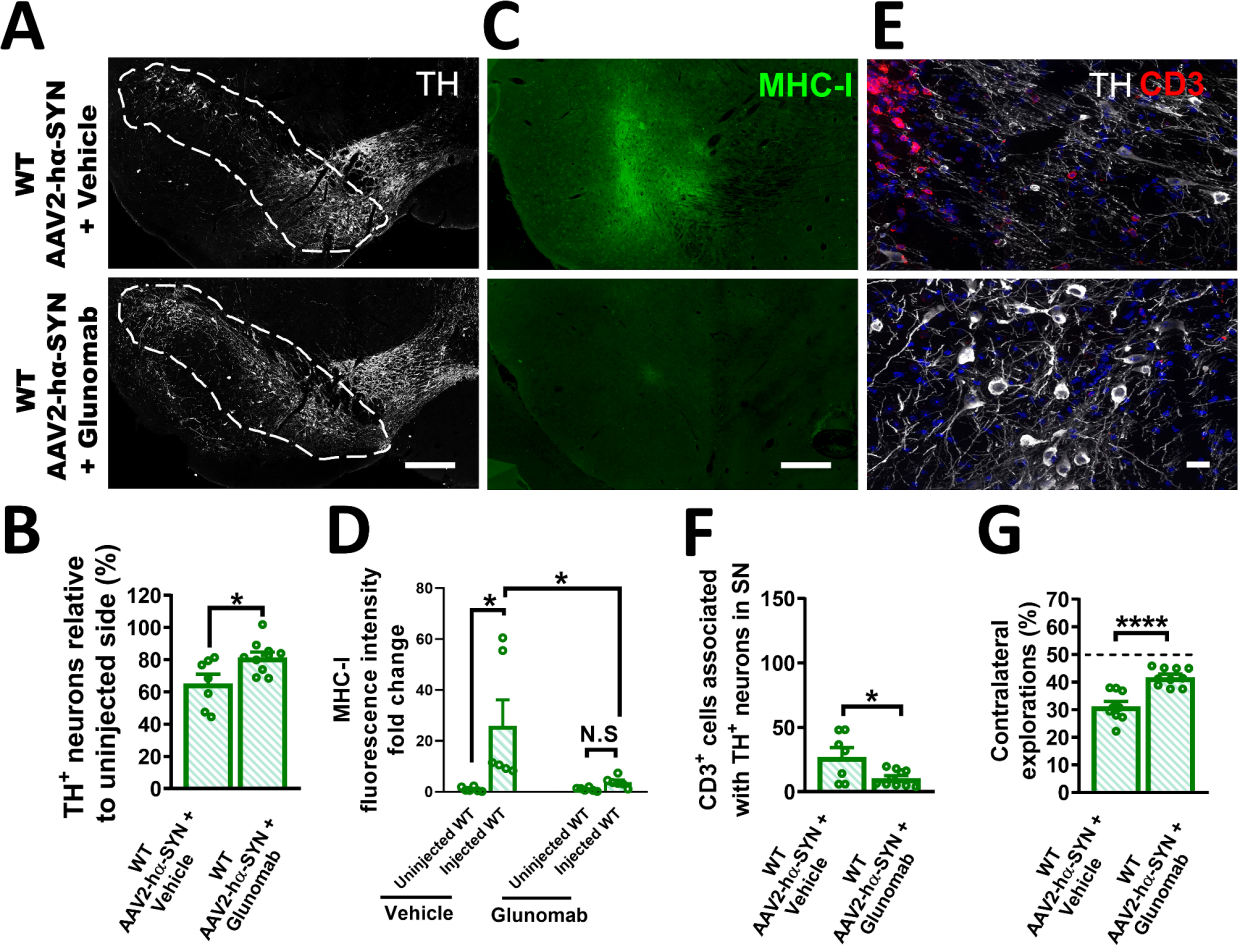
Intravenous delivery of glunomab reduces neuroinflammation and DA-neuron degeneration and rescues behavioral deficits after overexpression of hα-SYN. Representative images of the SN stained for (**A**) TH and (**C**) MHC-I, and the respective quantification of (**B**) DA-neuron survival and (**D**) MHC-I fluorescence intensity fold change in the SN 4 weeks after rAAV2-hα-SYN injection in WT mice treated with glunomab (10 mg/kg/week) or vehicle (*n* = 6–10). (**E**) Confocal image of the SN showing CD3^+^ cells (red) in contact with TH^+^ cell bodies and axons. (**F**) Quantification of CD3^+^ T cells associated with the SNpc 4 weeks after rAAV2-hα-SYN in WT mice treated with glunomab or vehicle (*n* = 7–10). (**G**) Quantification of sensorimotor bias in a corridor task 4 weeks after rAAV2-hα-SYN in WT mice treated with glunomab (10 mg/kg/week) or vehicle (*n* = 9–10). Glunomab treatment was performed on 12- and 16-week-old WT mice. For vehicle treatment, age-matched 12- and 16-week-old WT mice were used. Data are shown as mean ± SEM, N.S = not significant, *p < 0.05; ****p < 0.0001. **(B**,** F**,** G**) 2-tailed t-test; (**D**) 1-way ANOVA followed by Tukey post hoc test. Scale bar= **(A**,** C**) 250 μm (**E**) 20 μm

To test the therapeutic potential of inhibiting tPA-NMDAR1 binding in hα-SYN-mediated neurotoxicity, we treated WT mice intravenously with glunomab (10 mg/kg) or its respective vehicle. Mice were injected once a week for 4 weeks beginning 1 h after rAAV injection. Pharmacokinetic studies of glunomab in plasma and whole blood, indicated a glunomab half-life of ∼ 6 days (Fig. S9A). We found that intravenous glunomab treatment significantly protected DA-neurons (Fig. 8A, B) and reduced MHC-I levels and CD3 + T-cell infiltration (Fig. 8C-F) in the SN compared to vehicle after rAAV2-hα-SYN. The expression of hα-SYN in DA-neurons was confirmed in glunomab- and vehicle-treated WT mice (Fig. S9B). Consistent with the significant protection of DA-neurons and reduction of neuroinflammation by intravenous treatment, we observed a significant rescue in contralateral explorations in WT mice treated with glunomab compared to control (Fig. 8G).

## Discussion

In this study, we determined that striatal DARPP-32^+^ GABAergic neurons send tPA^+^ projections to the SN proximal to DA-neuron cell bodies and axons. We also demonstrated tPA involvement in DA-neuron degeneration in the context of hα-SYN-mediated neurotoxicity. Neuronal degeneration was associated with tPA-mediated upregulation of genes and proteins related to the innate and adaptive immune response in the SN. Furthermore, using two novel transgenic mouse models, we established that the effects of tPA were independent of its proteolytic activity. Lastly, we identified the NMDAR1 as a downstream effector of tPA-mediated DA-neuron degeneration using glunomab, an antibody that binds to NMDAR1 and blocks tPA binding to this receptor.

The physiological role of tPA in the SN is largely unknown. Since striatal DARPP-32^+^ GABAergic neurons express dopamine receptors and are known to respond to dopamine [44], the recognition that tPA is expressed in striatal GABAergic neurons and that tPA colocalizes with presynaptic axons innervating the SN suggests that tPA release in the SN may in part be regulated by dopamine signaling in striatal DARPP-32^+^ GABAergic neurons. This hypothesis is consistent with a study linking dopamine signaling in the striatum with increased tPA release in the nucleus accumbens [62]. Additionally, tPA may affect GABA signaling in the SN as inhibition of tPA reduces GABAergic synaptic activity in the hippocampus [63]; and we observed that tPA deficiency leads to baseline upregulation of the GABA-A receptor subunit *Gabra2* in the SN (Fig. 4A). Although tPA was observed in close proximity to the cell bodies and axons of dopaminergic neurons in the SN, further experiments using super-resolution or electron microscopy are required to confirm whether tPA forms synapses with these neurons.

While the physiological role of tPA in the SN is not known, our results provide evidence for the involvement of tPA in DA-neuron degeneration in the context of hα-SYN-mediated neurotoxicity (Fig. 3). A limitation in our study is that the source of tPA leading to neurodegeneration in the SN was not explored. We showed that the main source of tPA in the SN seems to be associated with presynaptic axons (Figs. 1 and 2), and overexpression of hα-SYN increased tPA immunoreactivity in the SN (Fig. 3). These results suggest that tPA released by neuronal terminals in response to hα-SYN neurotoxicity may be driving neuronal degeneration. However, tPA is also expressed by endothelial cells, and under some conditions, by microglia [64]; thus, the contribution of other cell populations to tPA release and DA-neuron degeneration cannot be ruled out.

To our knowledge, only one study has linked tPA with DA-neuron injury, where it was shown that injection of recombinant tPA directly into the SN leads to neuronal degeneration; however, tPA’s mechanism(s) of action and its involvement in PD were not studied [65]. Our data demonstrate that via a proteolytic-independent mechanism, endogenous tPA is needed for the upregulation of genes associated with the innate and adaptive immune system in response to overexpression of hα-SYN (Figs. 4, 5 and 6 and Fig. S7). We lack definitive evidence to confirm that tPA-mediated inflammation is the primary driver of DA neuron degeneration following hα-SYN overexpression. However, in virus-based mouse models of PD, neuroinflammation has been shown to precede advanced dopaminergic degeneration [66]. In PD mouse models and in humans, proinflammatory microglia and T-cell-mediated neurotoxicity are closely involved in neuronal cell death [20, 56]. For example, depletion of microglia is shown to protect DA-neurons in neurotoxin models of PD [67] and to partially suppress hα-SYN aggregation and propagation [68]. These data are consistent with the correlation between DA-neuron protection and downregulation of pro-inflammatory markers, like MHC-I and CD16/32, in microglia and other immune cells in tPA-KO mice after overexpression of hα-SYN (Figs. 4 and 5 and Fig. S5). Other groups have found that T-cells are involved in DA-neuron degeneration in virus-based models of PD. The contribution of CD8^+^ and CD4^+^ T cells in DA-neuron degeneration is complex, with some reports suggesting only CD4^+^ T cell involvement [69] and others suggesting a role for both populations of T-cells in virus-based models of PD [70]. In our model, infiltration of both CD4^+^ and CD8^+^ T-cells was observed in WT mice, whereas lack of tPA resulted in a significant reduction in the infiltration of T-cells. Overexpression of hα-SYN is shown to produce MHC-I and MHC-II receptors responsive to hα-SYN in mice [70]. Interestingly, we observed that hα-SYN-mediated MHC-I expression, primarily in microglia, correlated with tPA expression, since relative to WT mice, MHC-I expression was significantly reduced by deletion of tPA but was significantly increased in mice overexpressing tPA (Figs. 4 and 5 and Fig. S7). Indeed, others have reported that MHC-I in microglia is necessary for CD8^+^ T-cell cytotoxicity in the brain [54]. These data suggest that tPA could be directly or indirectly involved in regulating CD8^+^ T-cells in the SN by promoting MHC-I expression in the context of hα-SYN-mediated damage. However, the role of tPA in CD4^+^ and CD8^+^ T cell-mediated cytotoxicity in PD pathology needs further investigation. Future research should explore whether tPA directly influences immune cells, such as microglia or T-cells as a mechanism contributing to neuroinflammation, or if it acts directly on neurons leading to secondary effects driving neuroinflammation. Furthermore, our findings should be validated in other PD mouse models, including those utilizing α-SYN preformed fibrils.

Using glunomab to block tPA-NMDAR1 binding, we found that the tPA-NMDAR1 interaction was necessary to induce DA-neuron degeneration in WT and tPA Ala-BAC mice subjected to rAAV2-hα-SYN injection. The mechanism whereby the tPA-NMDAR1 interaction promotes DA-neuron degeneration is not known, and data from published studies have suggested alternatively that the tPA-NMDAR1 interaction can either directly induce neurodegeneration via excitotoxicity or enhance neuroinflammation by facilitating immune cell infiltration. Support for the former hypothesis is mainly based on cortical neuron cultures showing that tPA’s non-proteolytic interaction with extrasynaptic NMDAR1 leads to excitotoxicity and neuronal cell death [41]. Consistent with this, we observed that DA-neurons express NMDAR1 and that blocking tPA’s interaction with NMDAR1 in the SN using intranigral glunomab protected against DA-neuron degeneration without affecting C1q or CD16/32 immunoreactivity in the SN (Fig. 7 and Fig. S8). Furthermore, recent studies demonstrated that hα-SYN expression in primary neurons increased extrasynaptic NMDAR activity contributing to synaptic loss [71], and in a mouse model of Huntington’s disease, extrasynaptic NMDAR activity is known to induce neuronal degeneration [72]. In our PD model, the tPA-NMDAR1 interaction in DA-neurons may be necessary to increase the extrasynaptic NMDAR activity that leads to neuronal degeneration [41, 71]. This initial DA-neuron damage may trigger the innate and adaptive immune response in the SN inducing further DA-neuron death. Alternatively, support for the tPA-NMDAR1 interaction primarily regulating neuroinflammation comes from observations in a mouse model of multiple sclerosis. In this model, tPA-NMDAR1 interaction in endothelial cells was suggested to enhance immune cell infiltration by facilitating blood-brain-barrier opening and worsening outcomes [28]. Indeed, our data show that intravenous glunomab treatment significantly reduced T-cells infiltration in the SN and protected DA-neurons (Fig. 8). These results suggest that tPA interaction with a systemic source of NMDAR1, possibly endothelial cells, could enhance T-cell infiltration, a step that has been suggested to be necessary for DA-neuron degeneration in hα-SYN models of PD [69, 70]. Regardless of whether the tPA-NMDAR1 interaction leads to neurodegeneration via either or both mechanisms, our data describe a novel pathway leading to DA-neuron degeneration in the context of overexpression of α-synuclein. The identification of this pathway suggests a new therapeutic target for neurodegenerative disorders such as PD. Therefore, we are in the process of developing a humanized version of glunomab, LYS241, for future human clinical use. Preliminary data show LYS241 has high affinity for the amino-terminal domain of the human NMDAR1, and continued optimization of LYS241 will allow us to explore its potential as novel therapeutic approach for the treatment of PD.

## Conclusions

In this study, we demonstrated that tPA is expressed and released by GABAergic projections in the SN. We further showed that tPA drives neuroinflammation and neurodegeneration in an α-SYN mouse model of PD through a non-proteolytic interaction with NMDAR1. By using Glunomab, an antibody that prevents NMDAR1 interaction with tPA without disrupting NMDAR1 ion channel function, we identified a potential therapeutic approach to inhibit tPA-mediated neuroinflammation and neurodegeneration in PD.

## Electronic supplementary material

Below is the link to the electronic supplementary material.

Supplementary Material 1

Supplementary Material 2

## Data Availability

The RNA-seq data is available at EMBL-EBI repository with the accession number: E-MTAB-11567 (https://www.ebi.ac.uk/biostudies/arrayexpress/studies/E-MTAB-11567). Data and unique resources reported in this paper will be shared by the appropriate lead contact upon request.

## Abbreviations

α-SYN: α-synuclein
aCSF: Artificial cerebral spinal fluid
BAC: Bacterial artificial chromosome
C1q: Complement-1 q
CBA: Chicken-beta actin
CNS: Central nervous system
CTB: Cholera toxin-B
DA: Dopaminergic
DARPP-32: Dopamine- and cAMP-regulated neuronal phosphoprotein of molecular weight 32 kDa
MHC-I: Major histocompatibility complex class I
NMDAR: N-methyl-D-aspartate receptor
Nsp: Neuroserpin
PAI-1: Plasminogen activator inhibitor-1
PD: Parkinson’s disease
PSD-95: Postsynaptic density protein 95
rAAV2: Recombinant adeno-associated virus serotype 2
SN: Substantia nigra
SNpc: Substantia nigra pars compacta
TH: Tyrosine hydroxylase
tPA: Tissue plasminogen activator
VAMP-2: Vesicle-associated membrane protein 2
VGLUT: Vesicular glutamate transporter
