# Supplementary material for: The interaction of tPA with NMDAR1 drives neuroinflammation and neurodegeneration in α-synuclein-mediated neurotoxicity
Source: J Neuroinflammation. 2025 Jan 14;22:8. doi: 10.1186/s12974-025-03336-3 (PMC11731172; doi:10.1186/s12974-025-03336-3)
Supplement: Supplementary file 1 — Supplementary Material 1 [file 12974_2025_3336_MOESM1_ESM.pdf]

Fig. S1

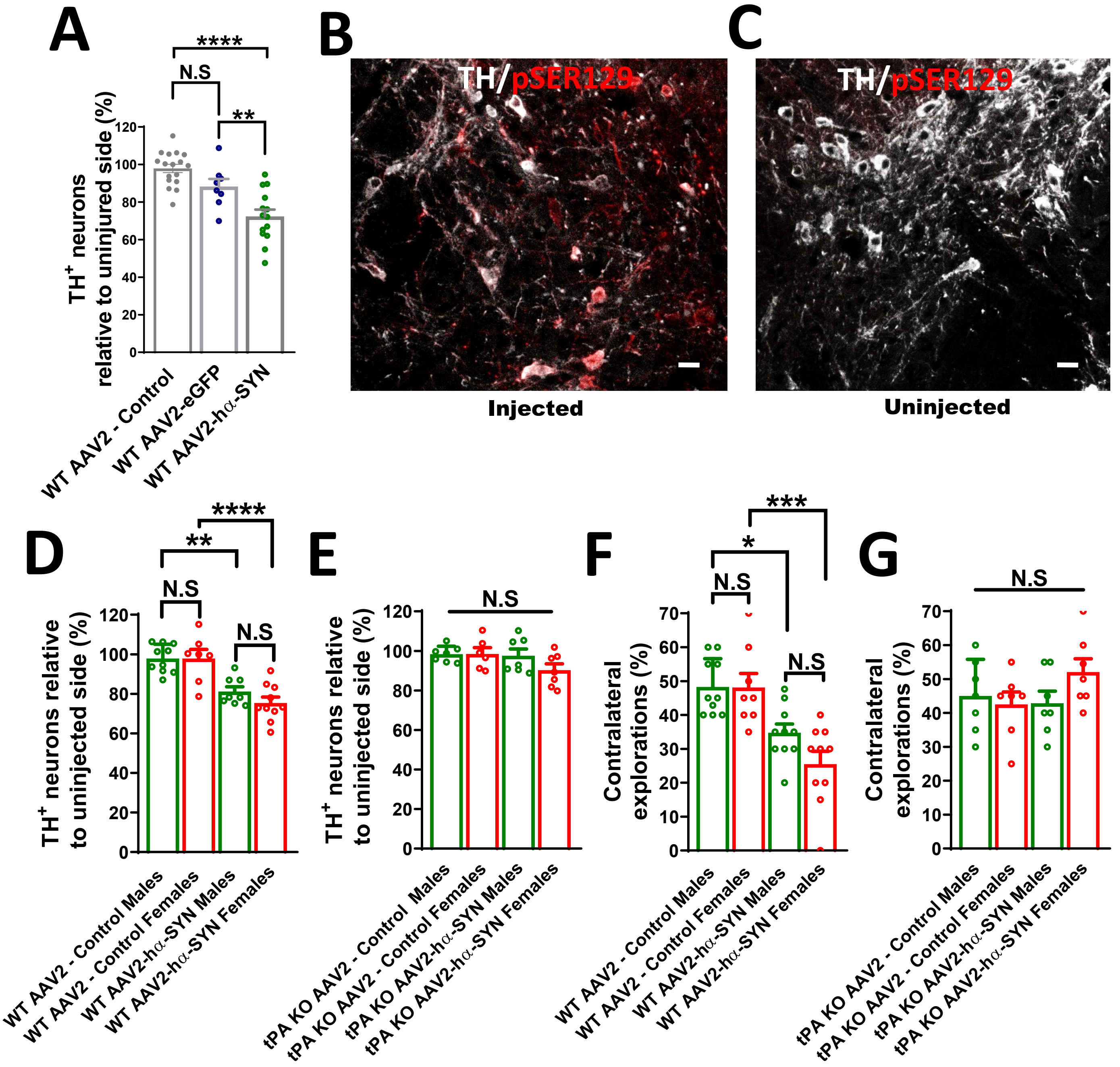
A
B
C
D
E
F
G
****
N.S
**
TH+ neurons relative to uninjured side (%)
WT AAV2 - Control
WT AAV2-eGFP
WT AAV2-hα-SYN
TH/pSER129
Injected
Uninjected
***
*
TH+ neurons relative to uninjected side (%)
Contralateral explorations (%)
WT AAV2 - Control Males
WT AAV2 - Control Females
WT AAV2-hα-SYN Males
WT AAV2-hα-SYN Females
tPA KO AAV2 - Control Males
tPA KO AAV2 - Control Females
tPA KO AAV2-hα-SYN Males
tPA KO AAV2-hα-SYN Females

Fig. S1: Characterization of the rAAV2-hα-SYN mouse model. (A) Quantification of TH+ neurons in coronal sections of the SN 4 weeks after rAAV2-hα-SYN, rAAV2-eGFP or rAAV2-empty (rAAV2-control) injection in WT mice (n=8-17); for this experiment, mice received rAAV2-hα-SYN or rAAV2-eGFP produced by the University of North Carolina at Chapel Hill Vector Core, obtained through The Michael J Fox Foundation; expression of hα-SYN was driven by a chicken-beta actin promoter (2 μL of the virus at a final concentration of 1.5 x 10^{13} vg/mL). **(B)** Representative image of the SN showing TH (white) and pS129-SYN (red) staining in the injected hemisphere of WT mice 4 weeks after unilateral injection of rAAV2-hα-SYN; (**C**) while no pS129-SYN signal was found in the uninjected hemisphere of the same section shown in **B**. (**D, E**) Quantification of TH+ neurons in coronal sections of the SN 4 weeks after rAAV2-hα-SYN or rAAV2-control injection in male and female WT and tPA-KO mice. (**F, G**) Quantification of sensorimotor bias in a corridor task 4 weeks after rAAV2-hα-SYN or rAAV2-control injection in male and female WT and tPA-KO mice (n=14-20). **(D, E, F, G)** Male and Female data were combined for final analysis and these data are presented in Figure 3C-G. These experiments were conducted using 9- to 11-week-old WT and tPA KO mice. Data are shown as mean ± SEM, N.S=not significant, *p<0.05; **p<0.01; ***p<0.001; ****p<0.0001. 1-way ANOVA followed by Tukey post hoc test. Scale bar= 20 μm.

Fig. S2

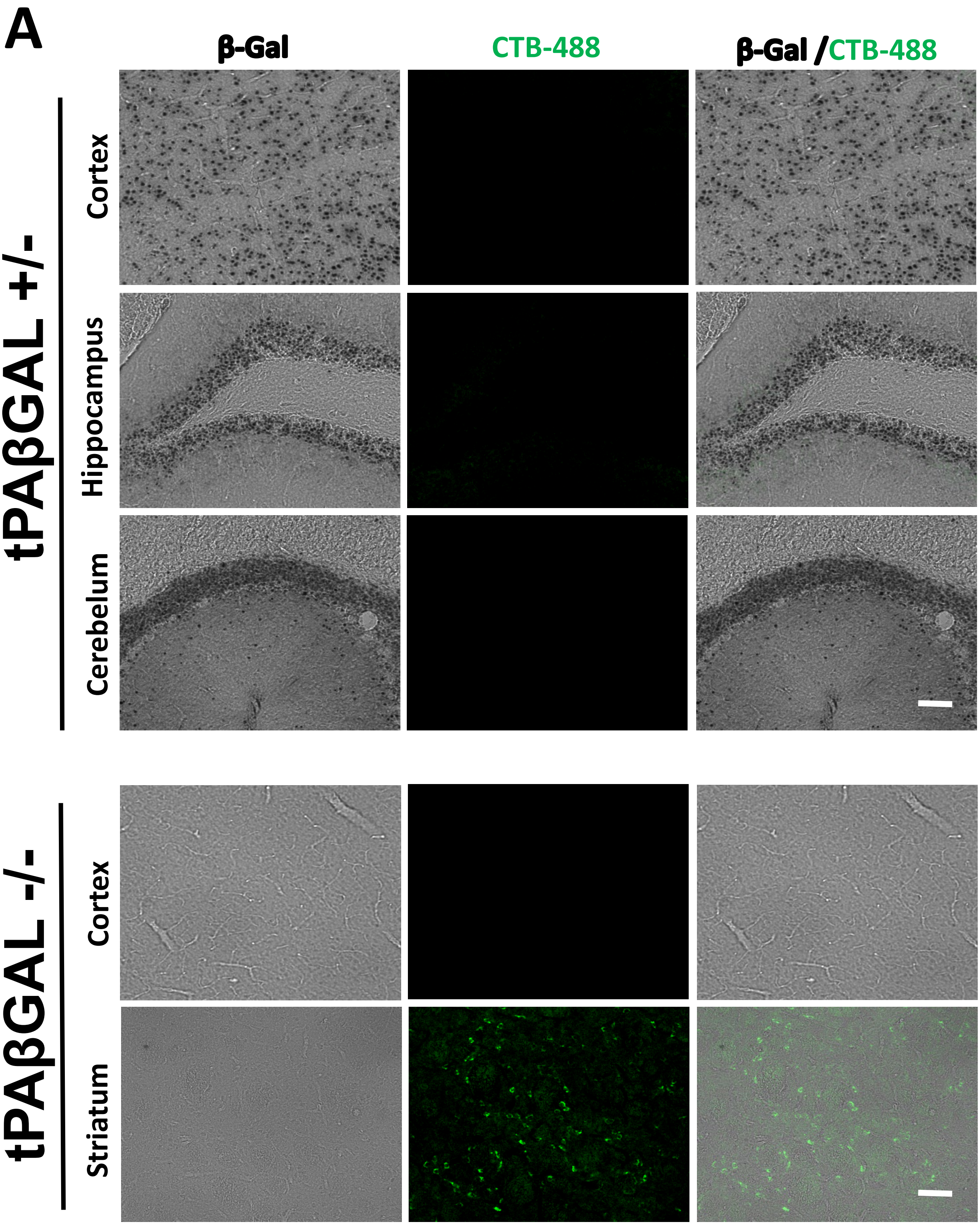
A
β-Gal
CTB-488
β-Gal /CTB-488
tPAβGAL +/-
Cortex
Hippocampus
Cerebelum
tPAβGAL -/-
Cortex
Striatum

Fig. S2: CTB-488+ neuronal cell bodies were only found in the striatum after CTB-488 injection in the SN. (A) Representative images of coronal sections of different brain regions known for tPA expression in neurons such as the cortex, hippocampus (dentate girus), and cerebellum showing β-Gal positive cells (black) but no CTB-488 staining detected in neuronal cell bodies (green) in tPAβGAL +/- mice 1 week after CTB-488 injection. tPAβGAL -/- was used as a negative control for β-Gal activity in the cortex and striatum, only the striatum showed CTB-488 positive staining in neurons (green; n=3). Scale bar= 50 µm.

Fig. S3

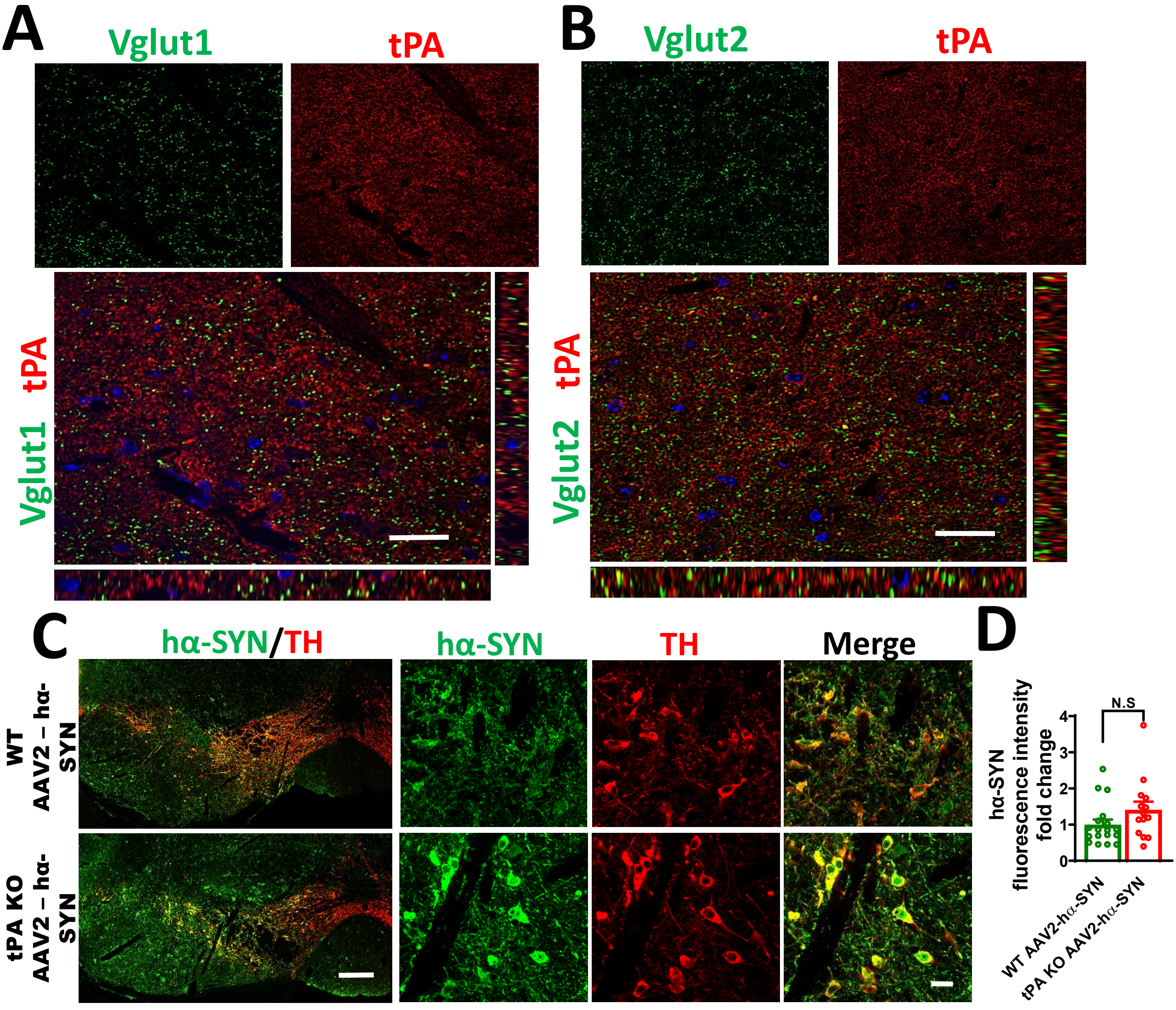

A
Vglut1
tPA
Vglut1 tPA
B
Vglut2
tPA
Vglut2 tPA
C
hα-SYN/TH
hα-SYN
TH
Merge
WT AAV2 – hα-SYN
tPA KO AAV2 – hα-SYN
D
hα-SYN fluorescence intensity fold change
N.S
0
1
2
3
4
WT AAV2-hα-SYN
tPA KO AAV2-hα-SYN

Fig. S3: hα-SYN was present in dopaminergic neurons of WT and tPA KO mice and overexpression of hα-SYN increased tPA immunoreactivity in the SN of WT mice. (A, B) Confocal images of the SN showing tPA (red) and Vglut1 or Vglut2 (green). Merged image (xy plane) shows low colocalization of tPA with Vglut1 or Vglut2. MOC calculation confirmed low colocation between tPA and Vgult1 (MOC= 0.127± 0.040; n=3) or Vglut2 (MOC=0.049± 0.012; n=3). Panels next to merge image (xy) show orthogonal view indicating xz (bottom panel) and yz (right panel) planes.

(C) Representative images of the SN showing TH (red) and hα-SYN (green) staining and close up showing hα-SYN expression in TH+ neurons in both WT and tPA KO mice (yellow). **(D)** Quantification of hα-SYN fluorescence intensity fold change relative to WT mice in the SNpc 4 weeks after rAAV2-hα-SYN injection (n=14-18). Data is shown as mean values ± SEM, N.S=not significant; 2-tailed t-test. Scale bar= (A, B and C close up) 20 μm (C) 250 μm.

Fig. S4

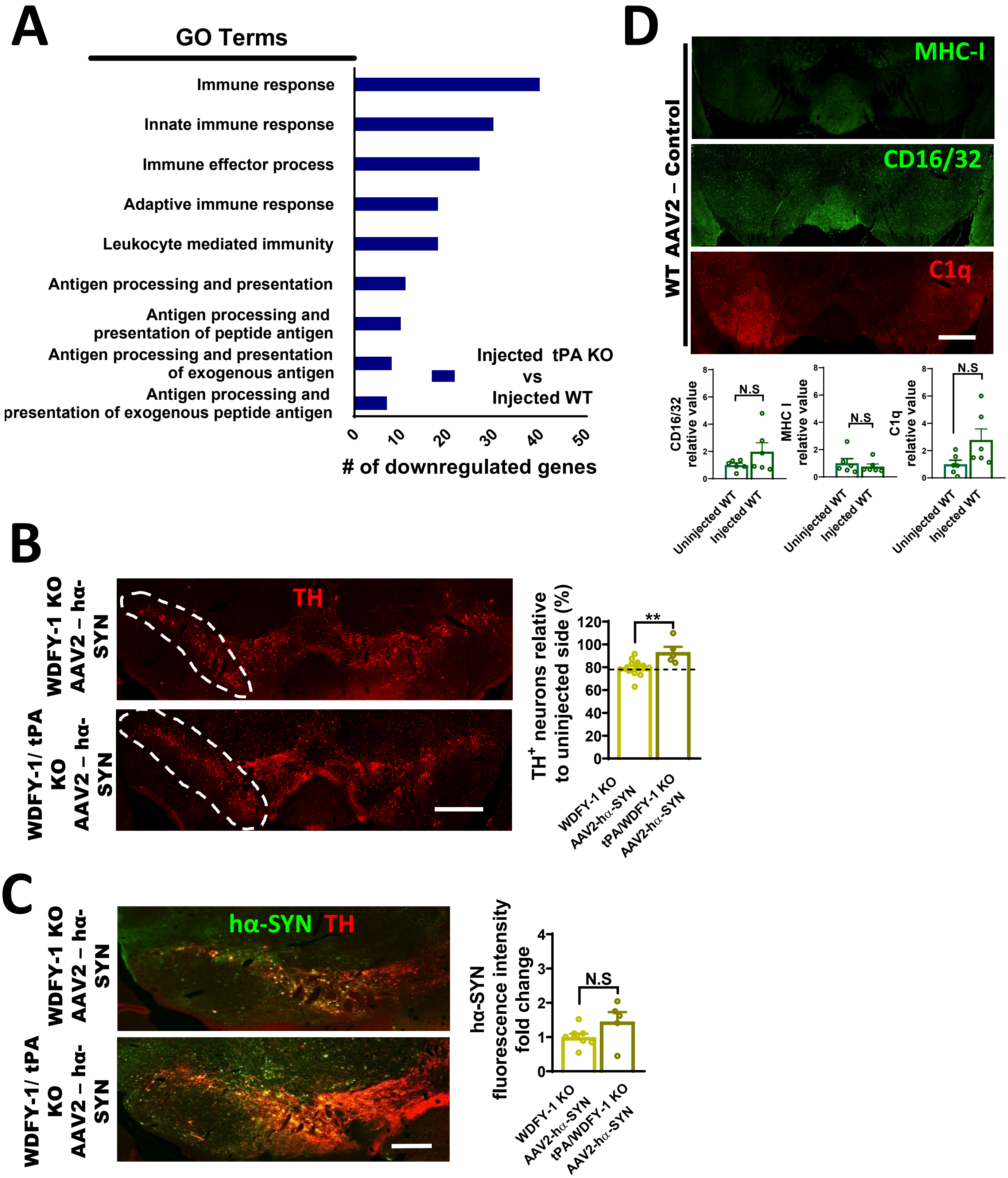

A
GO Terms
Immune response
Innate immune response
Immune effector process
Adaptive immune response
Leukocyte mediated immunity
Antigen processing and presentation
Antigen processing and presentation of peptide antigen
Antigen processing and presentation of exogenous antigen
Antigen processing and presentation of exogenous peptide antigen
Injected tPA KO vs Injected WT
# of downregulated genes
D
WT AAV2 – Control
MHC-I
CD16/32
C1q
CD16/32 relative value
MHC I relative value
C1q relative value
N.S
Uninjected WT
Injected WT
B
WDFY-1 KO AAV2 – hα-SYN
WDFY-1/ tPA KO AAV2 – hα-SYN
TH
TH+ neurons relative to uninjected side (%)
**
WDFY-1 KO AAV2-hα-SYN
tPA/WDFY-1 KO AAV2-hα-SYN
C
hα-SYN TH
hα-SYN fluorescence intensity fold change
N.S

Fig. S4: GO terms downregulated in tPA KO mice after rAAV2-hα-SYN injection, no involvement of WDFY-1 in dopaminergic neuron degeneration, and no significant effect of AAV2-control virus injection in pro-inflammatory markers in WT mice. (A) Top enriched GO terms from downregulated genes in the injected SN of tPA KO mice compared to the injected SN of WT mice 4 weeks after rAAV2-hα-SYN injection. All genes with a fold change > |1.5| (log2FC > |0.58|) were considered for this analysis. The x-axis represents the number of genes downregulated in tPA KO (n=4).

(B) Representative images of the SN showing dopaminergic neurons degeneration in the injected SN (TH, red) and quantification of TH+ neurons in coronal sections of the SN 4 weeks after rAAV2-hα-SYN injection in WDFY-1 KO and WDFY-1/tPA KO mice (black dashed line represent the historical average of dopaminergic neuron survival in WT mice 4 weeks after rAAV2-hα-SYN injection (n=5-8). **(C)** Representative images of the SN showing TH (red) and hα-SYN (green) staining in the SN confirming hα-SYN expression in TH+ neurons in both WDFY-1 KO and WDFY-1/tPA KO mice (yellow). Quantification of hα-SYN fluorescence intensity fold change relative to WDFY-1 KO mice in the SNpc 4 weeks after rAAV2-hα-SYN injection in the SN (n=5-8). (B-C) These experiments were conducted using 9- to 12-week-old WDFY-1 KO mice, and 10- to 13-week-old WDFY-1/tPA KO mice.

(D) Coronal sections of the SN showing MHC-I, CD16/32 and C1q staining and the respective quantification of fluorescence intensity fold change relative to the uninjected SN 4 weeks after rAAV2-control injection in WT mice. Injection of rAAV2-control virus in WT mice showed a trend in the increase of C1q but was not significant (p=0.066). This suggests that C1q upregulation might be in part associated with the injection or virus insult and not with hα-SYN-mediated neurotoxicity. White dashed lines represent the SNpc (n=6). Data is shown as mean values ± SEM, N.S=not significant, **p<0.01; 2-tailed t-test. Scale bar= (B, D) 500 µm (C) 250 µm.

Fig. S5

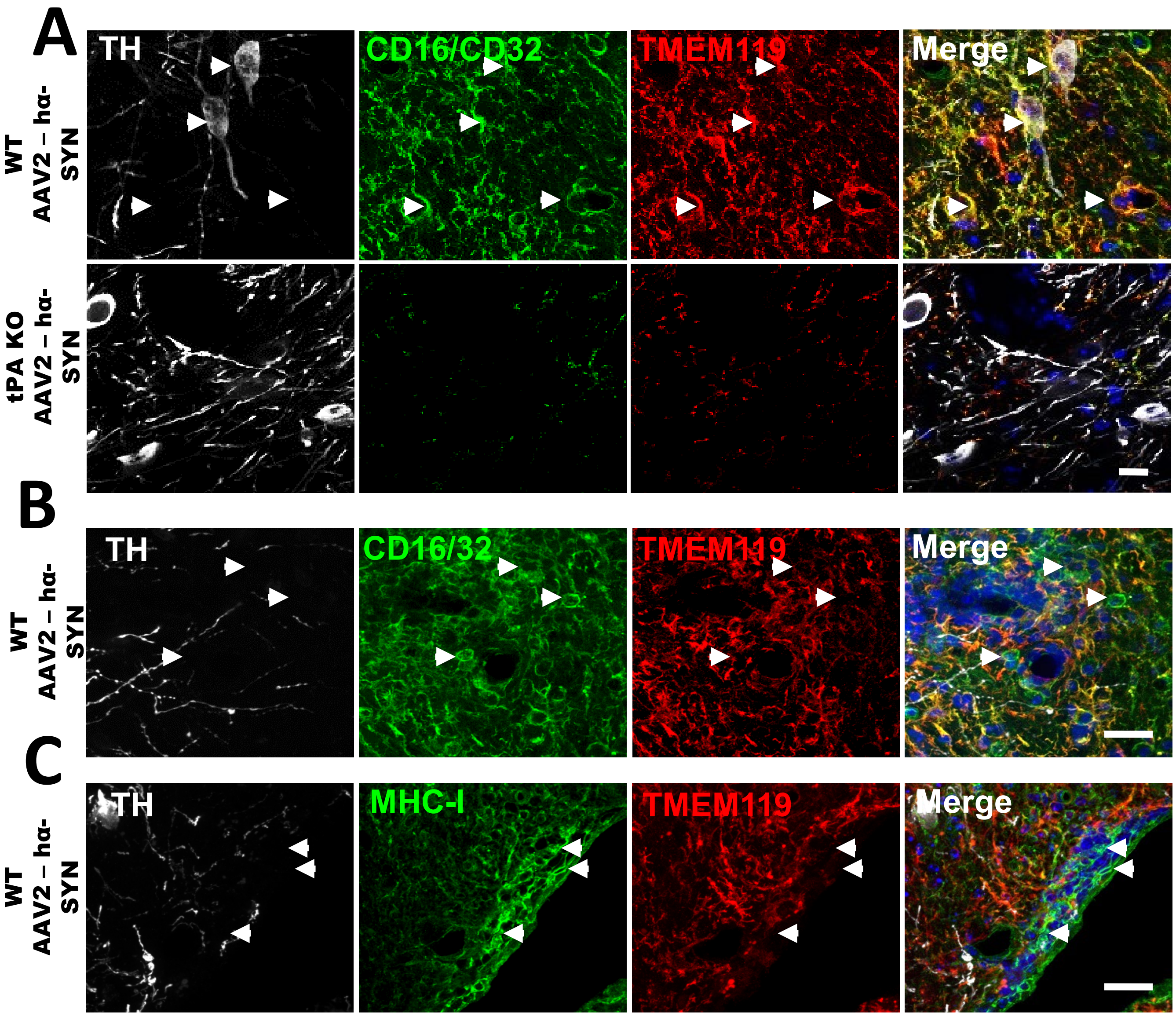
A
TH
CD16/CD32
TMEM119
Merge
WT AAV2 – hα-SYN
tPA KO AAV2 – hα-SYN
B
TH
CD16/32
TMEM119
Merge
WT AAV2 – hα-SYN
C
TH
MHC-I
TMEM119
Merge
WT AAV2 – hα-SYN

Fig. S5: CD16/32+ microglia are in contact with dopaminergic neurons of WT mice after overexpression of hα-SYN; Cells close to blood vessels are MHC-I+ or CD16/32+ but TMEM119- in WT mice after AAV2-hα-SYN injection. (A) Confocal images of the SN showing TMEM119 (red), TH (white) and CD16/32 (green) staining 4 weeks after rAAV2-hα-SYN injection in WT and tPA KO mice. CD16/32 colocalize with TMEM119 in WT mice after rAAV2-hα-SYN injection (MOC= 0.86 ± 0.01; n=3). Arrows indicate CD16/32(+) and TMEM119(+) cells near dopaminergic neuron cell bodies and axons (n=3).

(B) Confocal images of the SN showing TMEM119 (red), TH (white) and CD16/32 (green) or **(C)** TMEM119 (red), TH (white) and MHC-I (green) staining 4 weeks after rAAV2-hα-SYN injection in WT mice. Arrows indicate (B) CD16/32(+) and TMEM119(-) cells near blood vessels or (C) MHC-I(+) and TMEM119(-) cells near a blood vessel (n=3). Scale bar= (A) 10 μm (B-C) 20 μm.

Fig. S6

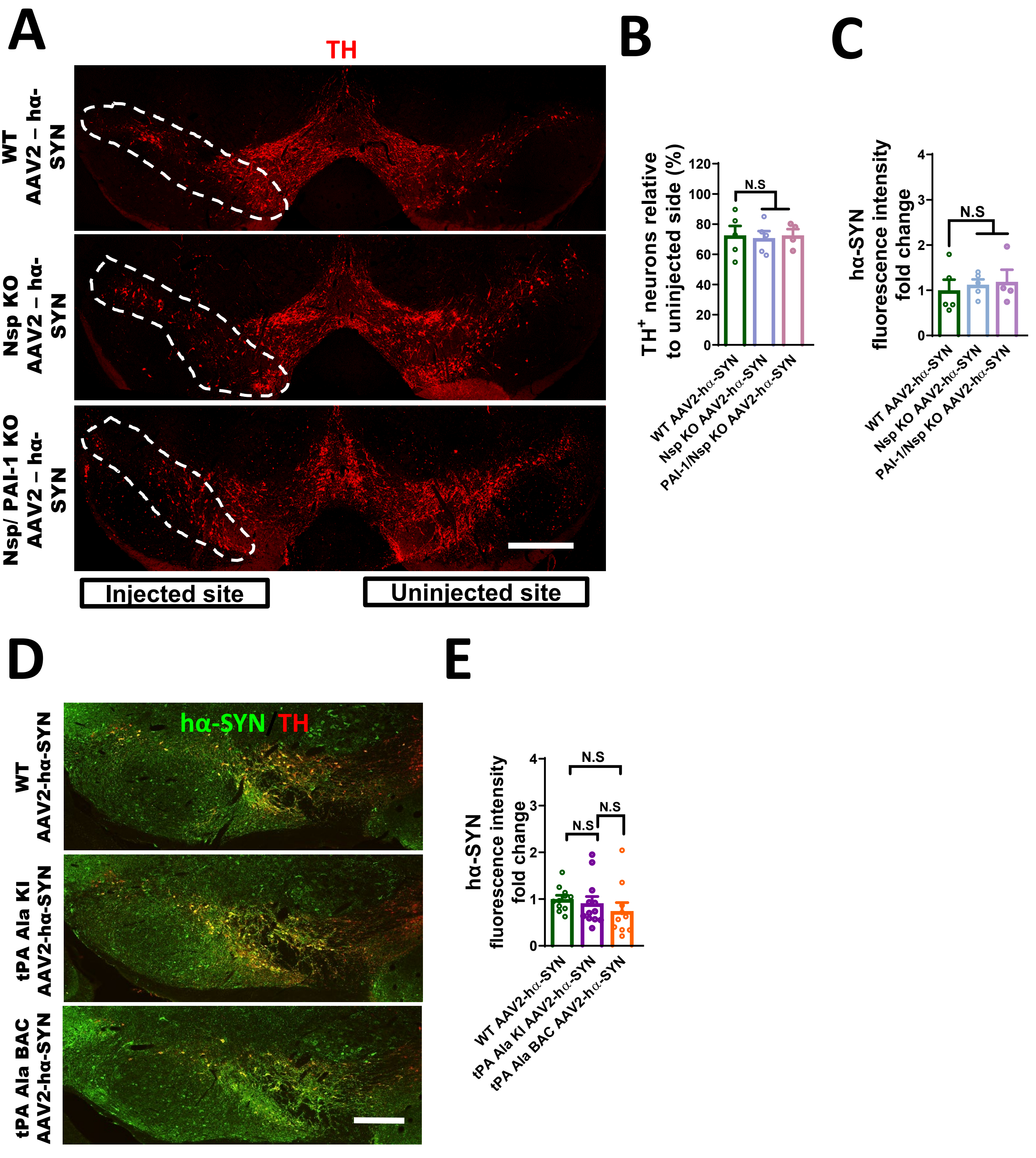
A
TH
WT AAV2 – hα-SYN
Nsp KO AAV2 – hα-SYN
Nsp/ PAI-1 KO AAV2 – hα-SYN
Injected site
Uninjected site
B
TH+ neurons relative to uninjected side (%)
N.S
WT AAV2-hα-SYN
Nsp KO AAV2-hα-SYN
PAI-1/Nsp KO AAV2-hα-SYN
C
hα-SYN fluorescence intensity fold change
N.S
WT AAV2-hα-SYN
Nsp KO AAV2-hα-SYN
PAI-1/Nsp KO AAV2-hα-SYN
D
hα-SYN/TH
WT AAV2-hα-SYN
tPA Ala KI AAV2-hα-SYN
tPA Ala BAC AAV2-hα-SYN
E
hα-SYN fluorescence intensity fold change
N.S
N.S
N.S
WT AAV2-hα-SYN
tPA Ala KI AAV2-hα-SYN
tPA Ala BAC AAV2-hα-SYN

Fig. S6: tPA inhibitors, Nsp and PAI-1, are not involved in dopaminergic neuron degeneration after rAAV2-hα-SYN injection. (A) Representative images of the SN showing dopaminergic neuron degeneration in the injected SN (TH, red).

(B) Quantification of TH+ neurons and **(C)** hα-SYN fluorescence intensity fold change relative to WT mice in coronal sections of the SNpc 4 weeks after rAAV2-hα-SYN injection in WT, Nsp KO and Nsp/PAI-1 KO mice. For this experiment, all mice received a rAAV2-hα-SYN produced by the University of North Carolina at Chapel Hill Vector Core, obtained through The Michael J Fox Foundation; expression of hα-SYN was driven by a chicken-beta actin promoter (2 µL of the virus at a final concentration of 1.5 x 10^{13} vg/mL was injected; n=4-5). These experiments were conducted using 15-week-old WT mice, 12- to 14-week-old Nsp KO mice, and 8- to 15-week-old Nsp/PAI-1 KO mice.

(D) Representative images of the SN showing TH (red) and hα-SYN (green) staining in the SN confirming hα-SYN expression in TH+ neurons in WT, tPA Ala KI and tPA Ala BAC mice.

(E) Quantification of hα-SYN fluorescence intensity fold change relative to WT mice in the SNpc 4 weeks after rAAV2-hα-SYN injection (n=10-12). Data is shown as mean values ± SEM, N.S=not significant, 1-way ANOVA followed by Tukey post hoc test. Scale bar= (A) 500 µm (D) 250 µm.

Fig. S7

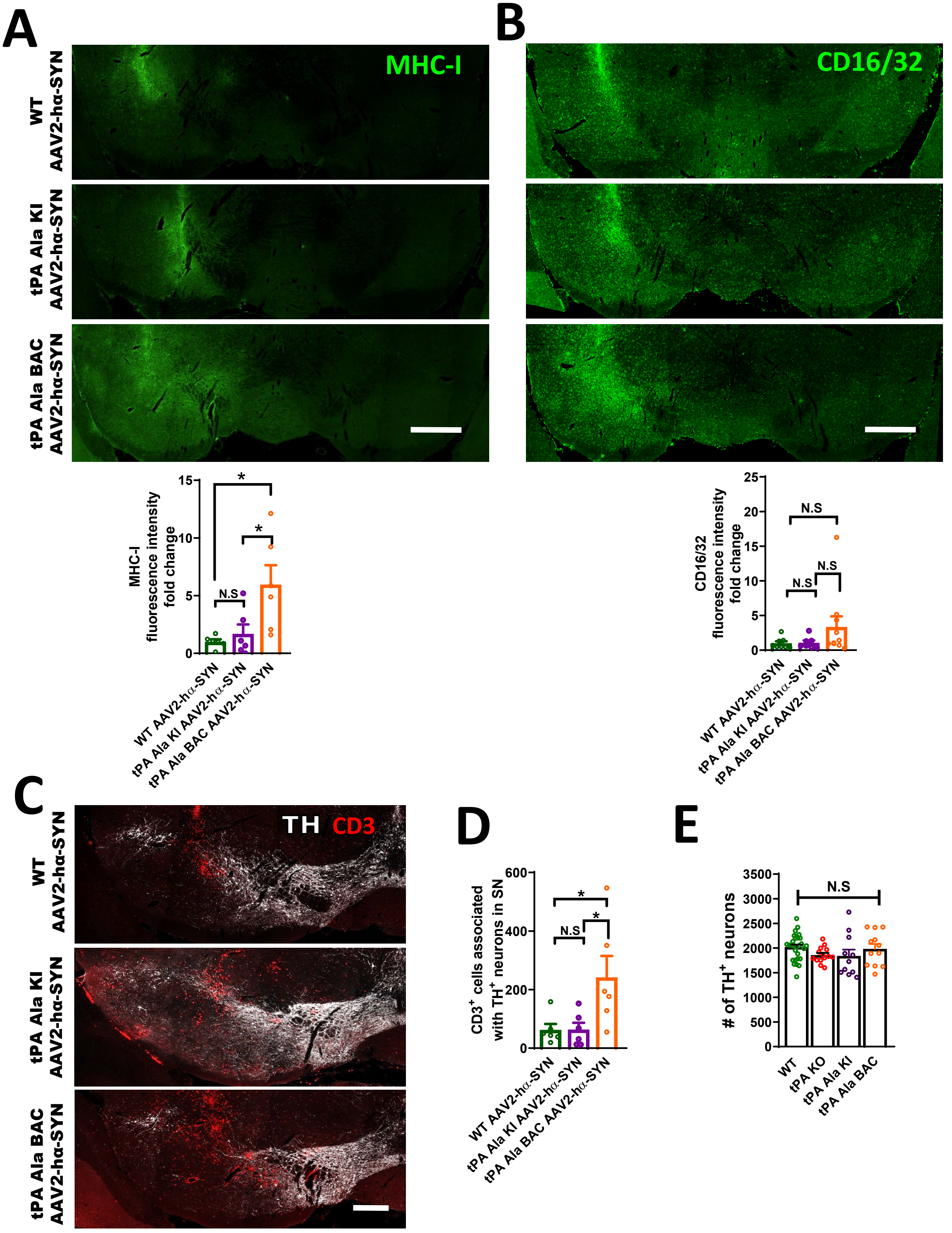
A
B
C
D
E
MHC-I
CD16/32
TH CD3
WT AAV2-hα-SYN
tPA Ala KI AAV2-hα-SYN
tPA Ala BAC AAV2-hα-SYN
MHC-I fluorescence intensity fold change
CD16/32 fluorescence intensity fold change
N.S
*
CD3+ cells associated with TH+ neurons in SN
# of TH+ neurons
WT
tPA KO
tPA Ala KI
tPA Ala BAC

Fig. S7: Proteolytically inactive tPA is able to induce a pro-inflammatory response in the SN after rAAV2-hα-SYN injection. Coronal sections of the SN showing **(A)** MHC-I and **(B)** CD16/32 staining and the respective quantification of fluorescence intensity fold change relative to WT mice 4 weeks after rAAV2-hα-SYN injection in WT, tPA Ala KI and tPA Ala BAC mice (n=6-10).
(C) Representative images of the SN showing CD3 (red) and TH (white) staining, and **(D)** quantification of CD3+ T cells associated with the SNpc 4 weeks after rAAV2-hα-SYN injection in WT, tPA Ala KI and tPA Ala BAC mice (n=6). **(E)** Quantification of total TH+ neurons in coronal sections of the uninjected SN 4 weeks after rAAV2-hα-SYN injection in WT, tPA KO, tPA Ala-KI and tPA Ala-BAC mice (n=11-29). Data is shown as mean values ± SEM, N.S=not significant, $*p<0.05$. (A, B, D) 1-way ANOVA followed by Tukey post hoc test. (E) Kruskal-Wallis test followed by Dunn's post hoc test. Scale bar= (A-B) 500 µm (C) 250 µm.

Fig. S8

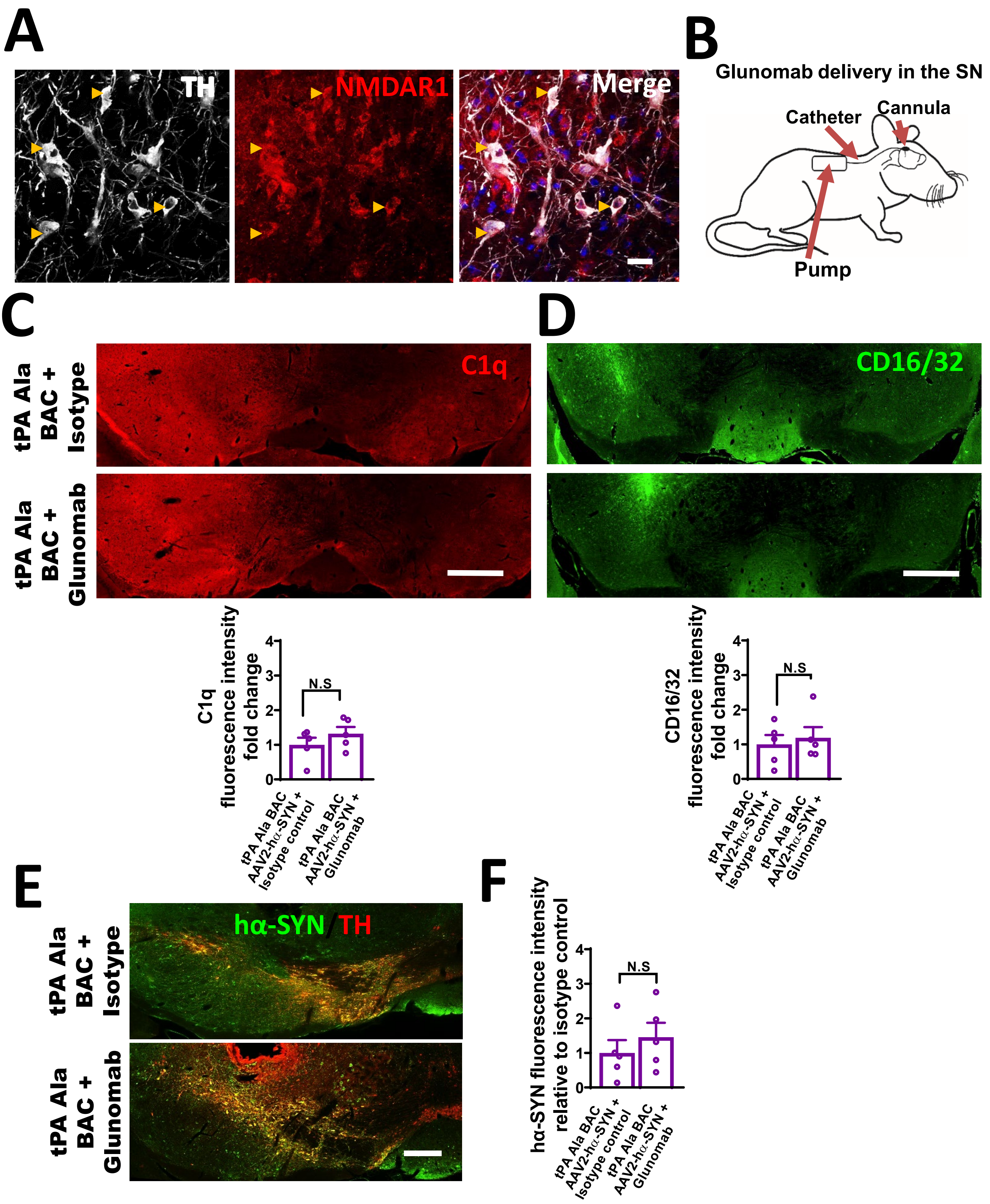
A
TH
NMDAR1
Merge
B
Glunomab delivery in the SN
Catheter
Cannula
Pump
C
tPA Ala BAC + Isotype
tPA Ala BAC + Glunomab
C1q
C1q fluorescence intensity fold change
N.S
tPA Ala BAC AAV2-hα-SYN + Isotype control
tPA Ala BAC AAV2-hα-SYN + Glunomab
D
CD16/32
CD16/32 fluorescence intensity fold change
N.S
tPA Ala BAC AAV2-hα-SYN + Isotype control
tPA Ala BAC AAV2-hα-SYN + Glunomab
E
tPA Ala BAC + Isotype
tPA Ala BAC + Glunomab
hα-SYN/TH
F
hα-SYN fluorescence intensity relative to isotype control
N.S
tPA Ala BAC AAV2-hα-SYN + Isotype control
tPA Ala BAC AAV2-hα-SYN + Glunomab

Fig. S8: NMDAR1 is expressed in dopaminergic neurons and Glunomab treatment did not reduce C1q or CD16/32 immunoreactivity in the SN after rAAV2-hα-SYN injection. (A) Confocal images of the SN showing TH (white) and NMDAR1 (red) staining in WT mice indicating NMDAR1 expression in dopaminergic neurons in the SNpc (arrows; n=3).

(B) Diagram of Glunomab delivery strategy into the SN using an osmotic pump after rAAV2-hα-SYN injection.

Representative images of the SN showing **(C)** C1q and **(D)** CD16/32 staining and the respective quantification of C1q and CD16/32 fluorescence intensity fold change relative to tPA Ala BAC Isotype treated mice 4 weeks after rAAV2-hα-SYN injection (n=5).

(E) Representative images of the SN showing TH (red) and hα-SYN (green) staining in the SN confirming hα-SYN expression in TH+ neurons (yellow) in tPA Ala BAC Isotype and Glunomab treated mice.

(F) Quantification of hα-SYN fluorescence intensity fold change relative to tPA Ala BAC Isotype treated mice 4 weeks after rAAV2-hα-SYN injection (n=5). Data is shown as mean values ± SEM, N.S=not significant. 2-tailed t-test. Scale bar= (A) 20um (C-D) 500 µm (E) 250 µm.

Fig. S9

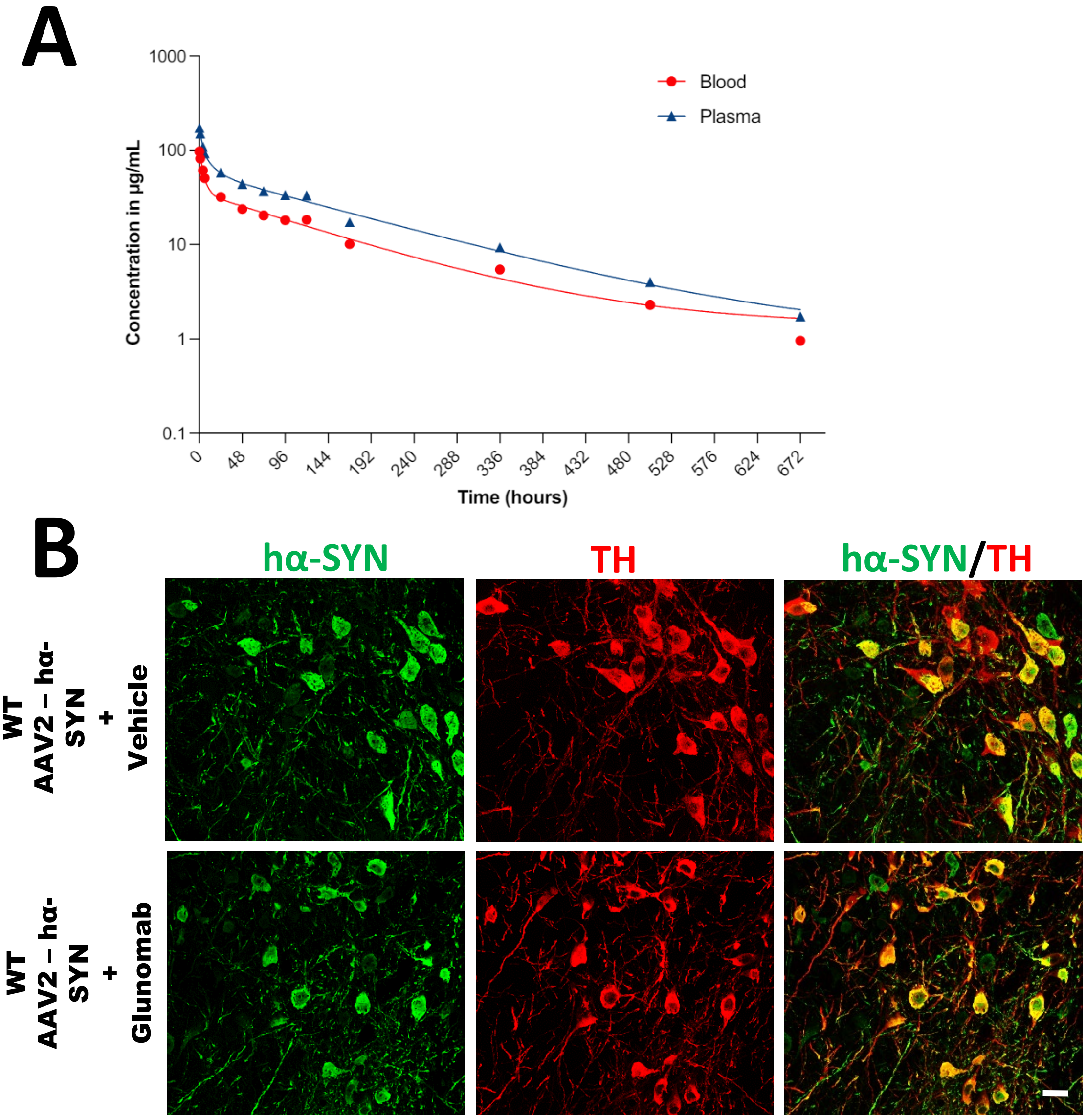

A
Blood
Plasma
Concentration in µg/mL
1000
100
10
1
0.1
0
48
96
144
192
240
288
336
384
432
480
528
576
624
672
Time (hours)
B
hα-SYN
TH
hα-SYN/TH
WT AAV2 – hα-SYN + Vehicle
WT AAV2 – hα-SYN + Glunomab

Fig. S9: Glunomab Pharmacokinetics and confirmation of hα-SYN expression in dopaminergic neurons in WT mice. (A) Blood and plasma levels of 125I radiolabel Glunomab at different timepoints (5 min, 1, 4, 6, 24, 48, 72, 96, 120, 168, 336, 504 and 672 h) after a single injection of Glunomab (10 mg/Kg) in CD-1 mice (n=3 per timepoint). Glunomab showed a bi-phasic decline and a mean terminal half-life of 150 hours (6.25 days) and mean residence time (MRT) of 179.63 (about 7.5 days).

(B) Representative confocal images of the SN showing TH (red) and hα-SYN (green) staining indicating hα-SYN expression in TH+ neurons in WT mice treated with Glunomab (10mg/kg/week) or vehicle in the SNpc 4 weeks after rAAV2-hα-SYN injection (n=9-10; yellow). Scale bar= 20 µm.
