# Supplementary material for: The interaction of tPA with NMDAR1 drives neuroinflammation and neurodegeneration in α-synuclein-mediated neurotoxicity
Source: J Neuroinflammation. 2025 Jan 14;22:8. doi: 10.1186/s12974-025-03336-3 (PMC11731172; doi:10.1186/s12974-025-03336-3)
Supplement: Supplementary file 2 — Supplementary Material 2 [file 12974_2025_3336_MOESM2_ESM.pdf]

Figure 1A

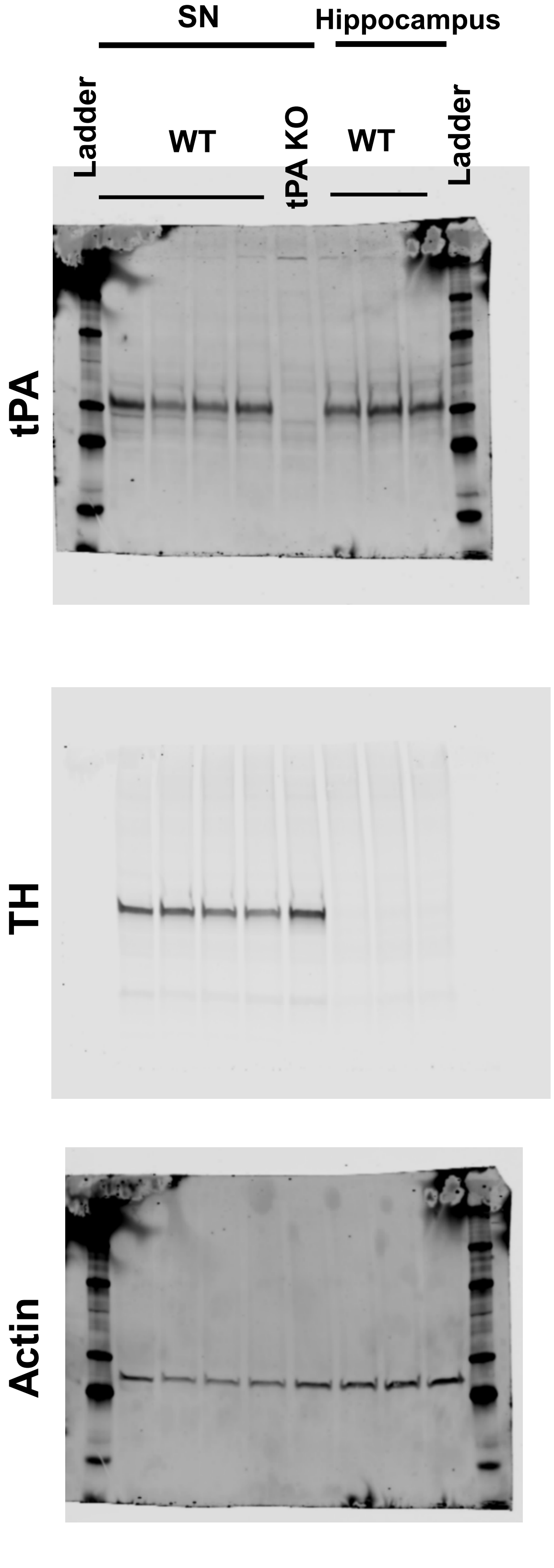

Figure 1B

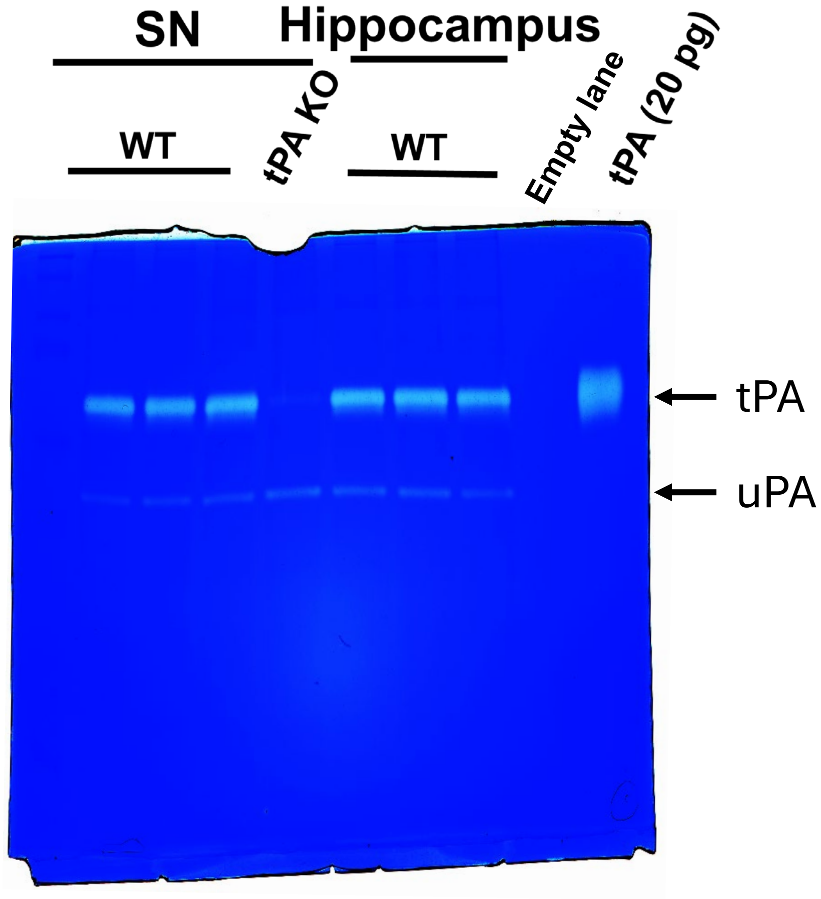

Figure 1D

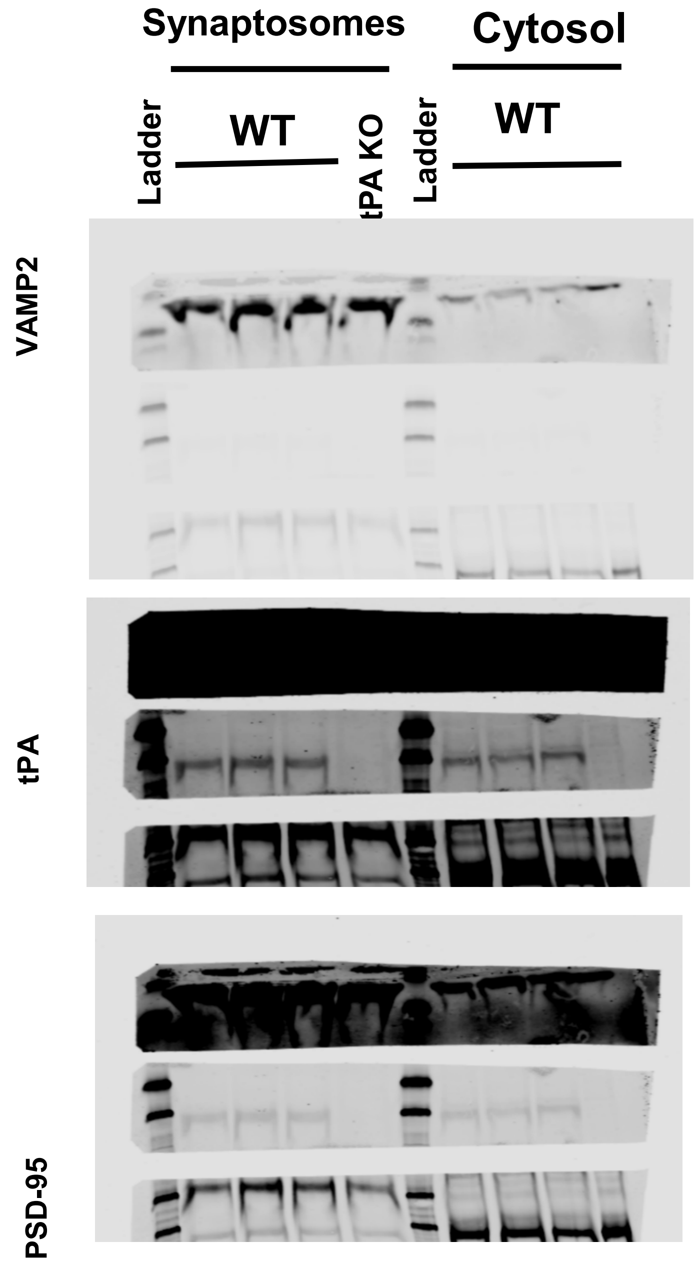

A single gel was run, then the membrane was cut for the development of proteins with different molecular weights using specific antibodies for either VAMP2, tPA, or PSD-95. Each specific antibody required different exposure times for detection. Each membrane strip is shown at each exposer.

Figure 1E

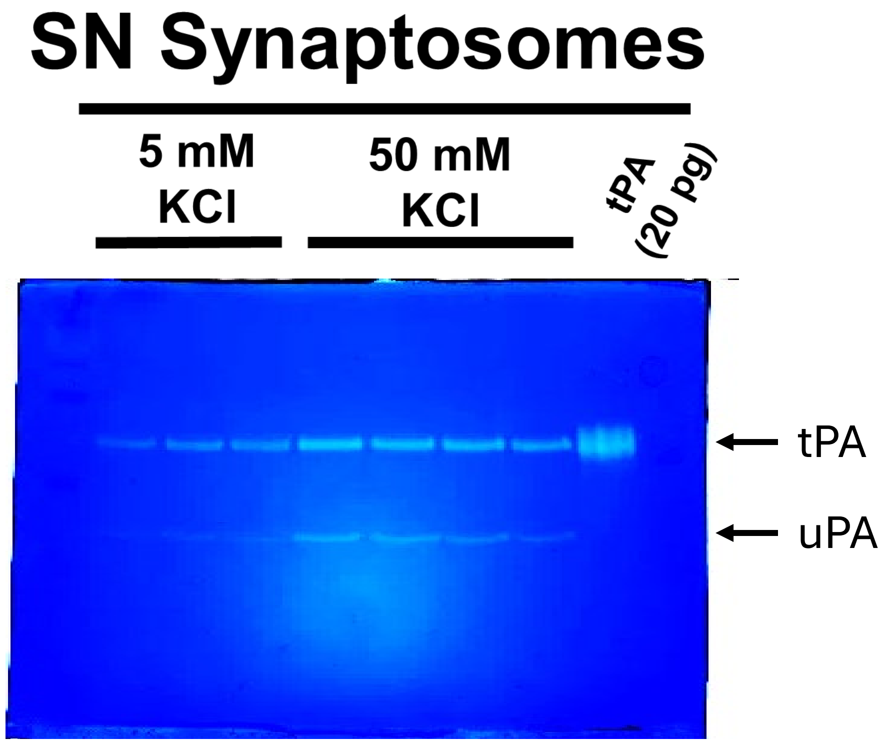

Figure 6B

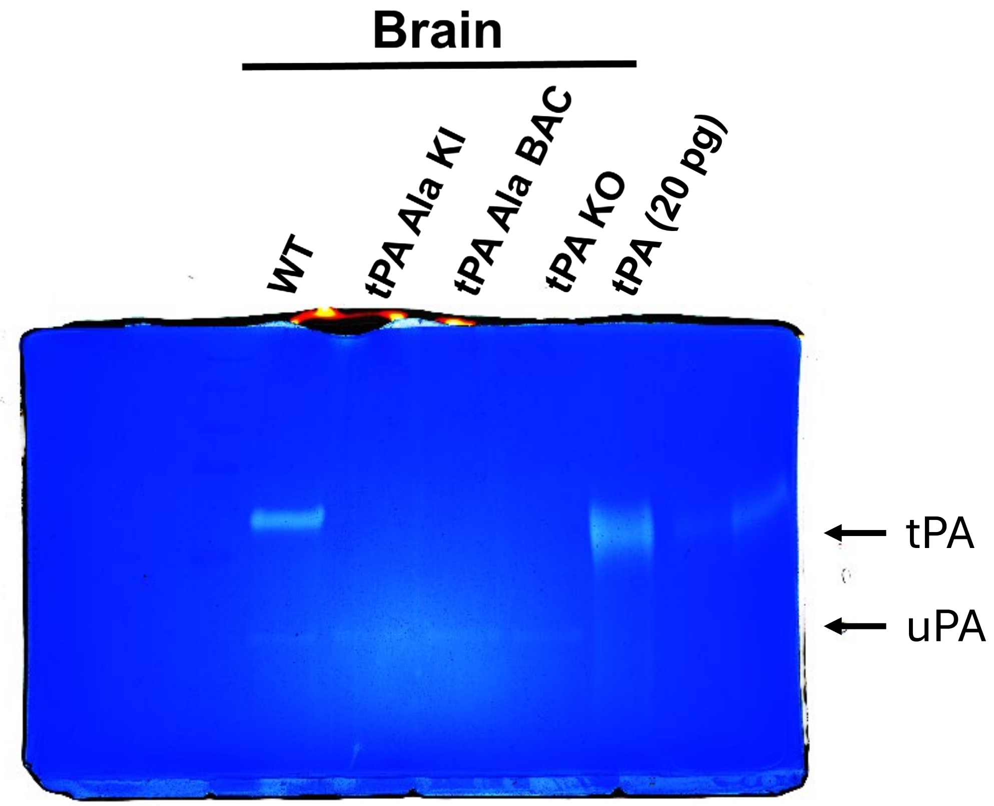

Brain
WT
tPA Ala KI
tPA Ala BAC
tPA KO
tPA (20 pg)
tPA
uPA

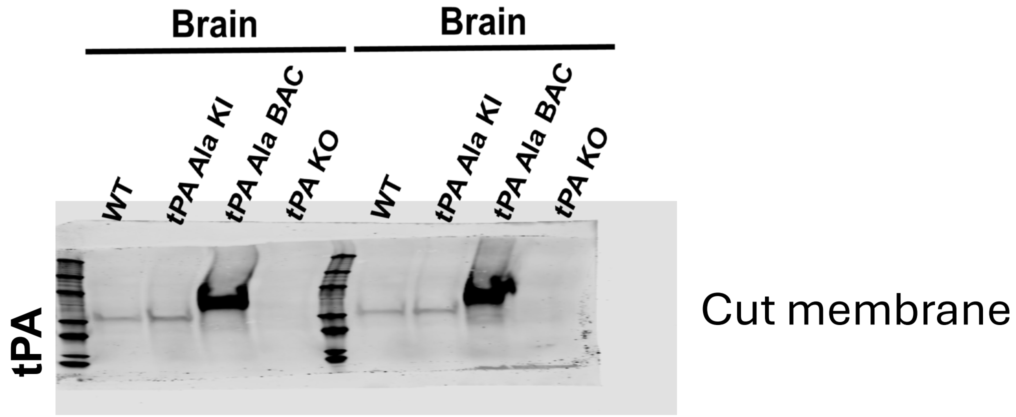

Brain
Brain
WT
tPA Ala KI
tPA Ala BAC
tPA KO
WT
tPA Ala KI
tPA Ala BAC
tPA KO
tPA
Cut membrane

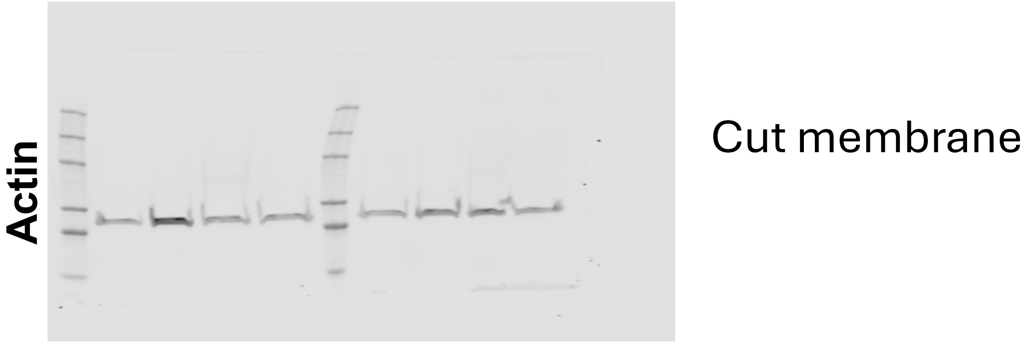

Actin
Cut membrane
